# Compound I Formation and Reactivity in Dimeric Chlorite Dismutase: Impact of pH and the Dynamics of the Catalytic Arginine

**DOI:** 10.1021/acs.biochem.2c00696

**Published:** 2023-01-27

**Authors:** Daniel Schmidt, Nikolaus Falb, Ilenia Serra, Marzia Bellei, Vera Pfanzagl, Stefan Hofbauer, Sabine Van Doorslaer, Gianantonio Battistuzzi, Paul G. Furtmüller, Christian Obinger

**Affiliations:** † Institute of Biochemistry, Department of Chemistry, University of Natural Resources and Life Sciences, Vienna, Muthgasse 18, A-1190Vienna, Austria; ‡ BIMEF Laboratory, Department of Chemistry, University of Antwerp, 2000Antwerp, Belgium; § Department of Life Sciences, University of Modena and Reggio Emilia, 41100Modena, Italy; ∥ Department of Chemistry and Geology, University of Modena and Reggio Emilia, 41100Modena, Italy

## Abstract

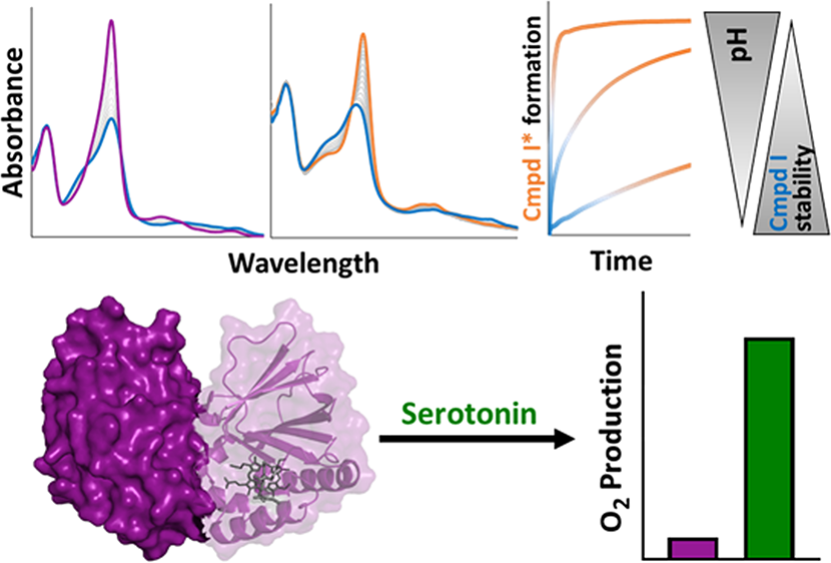

The heme enzyme chlorite dismutase (Cld) catalyzes the degradation of chlorite to chloride and dioxygen. Many questions about the molecular reaction mechanism of this iron protein have remained unanswered, including the electronic nature of the catalytically relevant oxoiron(IV) intermediate and its interaction with the distal, flexible, and catalytically active arginine. Here, we have investigated the dimeric Cld from *Cyanothece* sp. PCC7425 (*C*Cld) and two variants having the catalytic arginine R127 (i) hydrogen-bonded to glutamine Q74 (wild-type *C*Cld), (ii) arrested in a salt bridge with a glutamate (Q74E), or (iii) being fully flexible (Q74V). Presented stopped-flow spectroscopic studies demonstrate the initial and transient appearance of Compound I in the reaction between *C*Cld and chlorite at pH 5.0 and 7.0 and the dominance of spectral features of an oxoiron(IV) species (418, 528, and 551 nm) during most of the chlorite degradation period at neutral and alkaline pH. Arresting the R127 in a salt bridge delays chlorite decomposition, whereas increased flexibility accelerates the reaction. The dynamics of R127 does not affect the formation of Compound I mediated by hypochlorite but has an influence on Compound I stability, which decreases rapidly with increasing pH. The decrease in activity is accompanied by the formation of protein-based amino acid radicals. Compound I is demonstrated to oxidize iodide, chlorite, and serotonin but not hypochlorite. Serotonin is able to dampen oxidative damage and inactivation of *C*Cld at neutral and alkaline pH. Presented data are discussed with respect to the molecular mechanism of Cld and the pronounced pH dependence of chlorite degradation.

## Introduction

Chlorite is an angulate anionic [(p*K*_a_ (chlorous acid) = 1.96] and harmful oxidant [*E*°′(HClO_2_, 2H^+^/HOCl, H_2_O) = 1080 mV at pH 7.0, with *E*°′ increasing by 88 mV per pH unit with decreasing pH].^1^ The oxoanion is known to react with heme proteins in different ways. For example, it was shown to induce the formation of methemoglobin^2^ or to act as a hydroxylating agent in cytochrome P450.^3^ Horseradish peroxidase (HRP) mediates the heterolytic cleavage of chlorite and the two-electron oxidation of the ferric enzyme to Compound I [oxoiron(IV) porphyryl radical], thereby releasing hypochlorous acid that acts as a chlorinating agent.^4^ Furthermore, chlorite has been shown to act as a one-electron reductant of both Compound I and Compound II [oxoiron(IV)] of heme peroxidases, thereby forming chlorine dioxide.^4^ In contrast to HRP, chlorite cannot mediate the oxidation of the human peroxidases lactoperoxidase (LPO) or myeloperoxidase (MPO) to Compound I. These peroxidases are rapidly and irreversibly inactivated by heme bleaching and iron release, and the LPO/chlorite and MPO/chlorite systems do not mediate chlorination of target molecules.^5^

In case of the heme *b*-containing chlorite dismutases (Clds, EC 1.13.11.49),^6,7^ which belong to the structural superfamily of porphyrin-binding dimeric α + β barrel proteins,^8,9^ chlorite oxidizes the ferric state to an oxoiron(IV) intermediate that efficiently reacts with the transient chlorite reduction product, thereby releasing dioxygen (O_2_) and chloride (see below). This reactivity has not been observed in any other biological heme system to date. The exact mechanism is still under debate, and in principle, it might include the two-electron oxidation of ferric Cld to Compound I and heterolytic cleavage of chlorite to hypochlorite, as previously demonstrated with HRP (reaction 1).^4^ However, in contrast to HRP, in Cld, hypochlorite must then stay in the reaction sphere and rearrange, rebind, and react with Compound I to produce O_2_ and Cl^–^ (reaction 2). On the other hand, in case of homolytic cleavage of chlorite, Compound II and chlorine monoxide will be formed (reaction 3), again followed by rearrangement and reaction of these transient intermediates (reaction 4).
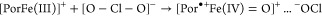
1
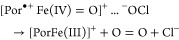
2
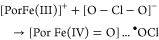
3
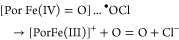
4

Many questions remain. Why can HRP^4^ but not LPO^5^ and MPO^5^ follow reaction 1? Are Clds able to catalyze the heterolytic cleavage of chlorite similar to HRP? Which discrepancies and peculiarities between peroxidases and chlorite dismutases are responsible for these significant different reactivities and the fact that reactions 2 or 4 and O_2_ generation are seen only in Clds?

**Figure 1:**
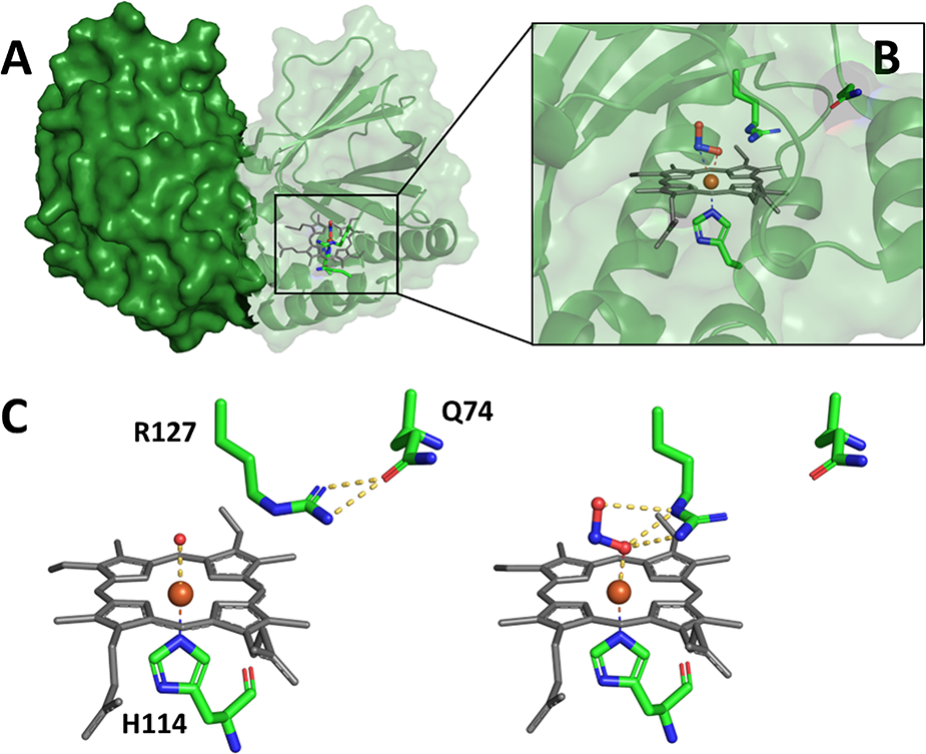
Crystal structure of wild-type chlorite dismutase from *Cyanothece* sp. PCC7425 (*C*Cld). (A) Overall crystal structure of homodimeric wild-type *C*Cld (PDB: 7OU5), with one subunit shown in surface representation and the other depicted in cartoon representation with a transparent surface representation in the background. (B) Active site architecture of the nitrite complex of *C*Cld showing the heme *b* cofactor (gray) in stick representation. The heme iron is depicted as an orange sphere, whereas the ligand nitrite is shown in stick and sphere representation. In addition, the proximal heme ligand H114 and the distal catalytic R127 in “in” conformation are depicted. (C) Comparison of the active site architecture of ferric native *C*Cld and its nitrite complex underlining the flexibility of the catalytic R127. In the resting state (left; PDB: 5MAU), water serves as the sixth heme ligand, and R127 is in the “out” conformation and hydrogen bonded to Q74. In the nitrite complex (right, PDB: 7OU5), this H-bond is broken, and R127 interacts with the anionic ligand. This figure was generated using PyMOL (http://www.pymol.org).

Both peroxidases^10^ and Clds^6,7^ have heme *b* (or posttranslationally modified heme) at the active site that is coordinated by a proximal histidine featuring a pronounced imidazolate character. In Clds, the proximal histidine (H114 in chlorite dismutase from *Cyanothece* sp. PCC7425, Figure 1) is hydrogen bonded to a conserved glutamate, whereas the proximal histidine in HRP is hydrogen bonded to an aspartate.^10^ This feature stabilizes the ferric resting state in both enzymes. The standard reduction potentials (*E*°′’s) of the redox couple Fe(III)/Fe(II) of these heme enzymes can have very different values falling around −116 mV in Clds^11^ and ranging from −310 mV in the heme *b* peroxidase HRP^12^ to −183 mV in LPO^13^ and up to +5 mV in MPO.^14^ In LPO and MPO, the positive *E*°′ values mainly reflect the posttranslational modifications of the prosthetic group.^10,15,16^ Thus, *E*°′[Fe(III)/Fe(II)] values of Clds are significantly more positive than those of heme *b* peroxidases and lie in between those of LPO and MPO (that cannot follow reaction 1).^5^ It has to be noted that dye-decolorizing peroxidases, which together with Clds belong to the same superfamily of porphyrin-binding dimeric α + β barrel proteins,^8,9^ exhibit *E*°′[Fe(III)/Fe(II)] values even more negative than HRP, *e.g.*, −350 mV for the B-class enzyme from *Klebsiella pneumonia*.^17,18^ These findings clearly indicate that, besides the imidazolate character of the proximal histidine and the posttranslational modification of the heme, the distal architecture also impacts the redox properties.

In Clds, the only charged amino acid in an otherwise hydrophobic distal cavity is a flexible catalytic arginine (R127 in *C*Cld, Figure 1), which switches between two conformations, *i.e.*, toward either the heme iron (“in”) or the substrate entry channel (“out”), depending on ligand binding, redox state, and pH.^19−27^ This fully conserved arginine has been the center of numerous structural and functional studies for its putative involvement in substrate recognition, binding, and conversion.^19−30^ Conformational mobility of a distal catalytic arginine is not limited to Clds; it has also been seen in heme peroxidases, *e.g*.*,* in HRP or cytochrome *c* peroxidase (C*c*P).^31,32^ In C*c*P, one position of R48 is “out” toward the heme propionates and a second “in” toward the heme iron.^31^ In case of Compound I of C*c*P [*i.e*., Compound I* in C*c*P, oxoiron(IV) Trp^•+^] and HRP, the distal arginine is exclusively in the “in” position.^31,32^ In addition to the distal arginine, peroxidases have either a histidine or an aspartate for heterolytic cleavage of hydrogen peroxide.^10^ The lack of a distal His or Asp base in Clds is consistent with their lack of catalase or (H_2_O_2_-mediated) peroxidase activity.^6,7^ As mentioned earlier, dye-decolorizing peroxidases are structurally most closely related to Clds (but lack Cld activity) and possess both distal Asp and Arg residues.^17,18^ However, their hemes in DyPs are flipped 180° in one and 90° in another direction in comparison to hemes of Clds, putting their distal arginine close to one of the propionic side chains and allowing the establishment of a salt bridge.^17^ Dye-decolorizing peroxidases cannot degrade chlorite to O_2_ and Cl^–^.

The crucial question is the following: Why is chlorite consumption in Clds always coupled to O_2_ generation, and why is chlorite consumption in all other heme systems, including peroxidases, always strictly uncoupled from O_2_ generation? Importantly, in all Cld arginine mutants studied so far, Cl–O bond cleavage is always coupled with O_2_ generation, even in those having the catalytic arginine exchanged by alanine, despite the fact that these variants exhibit very low activities and are prone to inactivation.^27,29,30^ This might suggest that the hydrophobic and sterically confined nature of the Cld distal pocket is important for protecting the reactive intermediates and prompting their conversion by a rebound mechanism.

The present paper aims at elucidating the impact of the dynamics of the conserved arginine R127 in the catalytic mechanism of the dimeric Cld from *Cyanothece* sp. PCC7425^33^ (Figure 1) by studying the reactions of the wild-type *C*Cld and the variants Q74V and Q74E with chlorite and hypochlorous acid in the pH range 5–9. Recently, the two variants have been shown to exhibit differences in the dynamics of R127 and, in turn, in heme coordination, ligand binding, and catalysis.^26,27^ The flexibility of R127 has been shown to follow the hierarchy Q74V > wild-type *C*Cld > Q74E. Here, we demonstrate by stopped-flow UV–vis spectroscopy that mixing of Cld and HOCl allows formation of pure *C*Cld Compound I. Hypochlorous acid is a stronger oxidant compared to chlorite [*E*°′(HOCl, H^+^/H_2_O, Cl^–^) = 1280 mV at pH 7.0]^34,35^ and known to convert heme peroxidases, including LPO and MPO, to pure Compound I.^36^ We show that Compound I of *C*Cld reacts with two- and one-electron donors like iodide, chlorite, and serotonin but not with hypochlorite. The stability of Compound I strongly depends on pH with pronounced formation of protein-based amino acid radicals at basic pH regimes. Importantly, we show that serotonin can partially prevent inhibition and oxidative damage of Cld. The findings are discussed with respect to the molecular mechanism of chlorite degradation and of the inhibition reaction, which becomes dominant in the alkaline pH range.

## Materials and Methods

### Protein Expression and Purification

Recombinant protein expression of wild-type *C*Cld and variants was performed in *Escherichia coli* BL21 Gold (DE3) cells (Agilent) in an LB medium supplemented with ampicillin as recently reported.^26^ For protein purification, the cell pellets were thawed and resuspended in a lysis buffer (50 mM phosphate buffer (pH 7.4), 500 mM NaCl, 0.5% Triton X-100, and 5% glycerol) supplemented with ∼100 μM hemin. After ultrasonication (two 3 min cycles, pulsed mode, 1 s sonication, 1 s rest, 90%) on ice, the cell lysate was centrifuged (4 °C, 17,000*g*, 35 min). Following a filtration step (0.45 μm, Durapore Membrane, Merck, Darmstadt, Germany), the resulting crude extract was loaded onto a His-trap affinity column (5 mL, GE Healthcare, Chicago, IL, USA) pre-equilibrated with a binding buffer (50 mM phosphate buffer (pH 7.4), 500 mM NaCl). The protein-loaded column was washed with the binding buffer, and cleavage of the His tag on the column was performed. For this purpose, the column was equilibrated with a cleavage buffer (50 mM Tris–HCl (pH 7.0) with 150 mM NaCl and 1 mM EDTA), and the cleavage with a His-tagged HRV 3C PreScission Protease was performed overnight at 4 °C. Elution was carried out with a storage buffer (50 mM phosphate buffer (pH 7.0)) accompanied by a concentration and desalting step using an Amicon Ultra-15 centrifugal filter unit (10 kDa molecular weight cutoff; Merck, Darmstadt, Germany). As a final polishing step, the concentrated protein was applied to a pre-equilibrated (50 mM phosphate buffer (pH 7.0)) HiLoad 16/60 Superdex 200 prep grade column (GE Healthcare). The collected fractions were pooled and concentrated to a concentration of ∼20 mg mL^–1^ using a centrifugal filter unit. Before being stored at −80 °C in 50–100 μL aliquots, the purified protein was analyzed by HPLC (Shimadzu Prominence LC20, Korneuburg, Austria) equipped with MALS (Wyatt Heleos Dawn 8+ QELS; software Astra 6, Dernbach, Germany), a refractive index detector (RID-10A; Shimadzu), and a diode array detector (SPD-M20A; Shimadzu) to determine the oligomerization state and purity.

### UV–Vis and Electronic Circular Dichroism (ECD) Spectroscopies

UV–vis spectra in a wavelength range between 200 and 800 nm were recorded at 25 °C using a Cary 60 UV–vis spectrophotometer (Agilent) and a U-3900 spectrophotometer (Hitachi, Mannheim, Germany) in 50 mM phosphate–citrate buffer (pH 5.0), 50 mM phosphate buffer (pH 7.0), and 50 mM phosphate–borate buffer (pH 9.0), respectively. The molar extinction coefficient of heme (ϵ_Soret_ = 100,000 M^–1^ cm^–1^) was used to determine the enzyme concentration.

Electronic circular dichroism (ECD) spectroscopy was performed using Chirascan (Applied Photophysics, Leatherhead, UK). Spectra were recorded in the visible region (260–500 nm). The path length of the used quartz cuvette was 10 mm. Conditions were as follows: 10 μM enzyme in 5 mM citrate–phosphate buffer (pH 5.0), 5 mM phosphate buffer (pH 7.0), or 5 mM borate–phosphate buffer (pH 9.0), respectively. Spectral bandwidth was 1 mm, and scan speed was 5 s nm^–1^.

### Electron Paramagnetic Resonance Spectroscopy (EPR)

In the attempt to trap the short-lived intermediates formed during the reaction of *C*Cld with hypochlorite (ClO^–^) and chlorite (ClO_2_^–^), samples for electron paramagnetic resonance (EPR) spectroscopy were prepared using a rapid freeze-quench (RFQ) device from BioLogic (Grenoble, France), consisting of an SFM-2000 stopped-flow unit and an MPS-70 controller unit, combined with a freeze-quench sample collector adapted for EPR tubes. The ejected volumes and flow rate were controlled by the BioLogic BIOKINE software, v. 4.72. This RFQ setup was calibrated by quenching the binding reaction of azide (N_3_^–^) to ferric myoglobin (Mb) at selected time points as described by Pievo *et al.*(37) (see the Supporting Information for a more detailed description of the procedure). The calibration showed that the shortest achievable freezing time was ∼50 ms (see Figure S4). Therefore, for all *C*Cld samples, the reaction was quenched at ∼60 ms, including the aging and the flying time of the reacting mixture that can be calculated from the chosen settings of the freezing experiment. In all cases, protein samples were prepared in 100 mM sodium phosphate–citrate buffer (pH 5), which was filtered and degassed prior use. For the reaction with hypochlorite, the samples were prepared in triplicate by mixing 50 μL of a solution containing *C*Cld at a concentration of 0.5 mM with 50 μL of a 5 mM sodium hypochlorite (NaClO) solution prepared in 1 mM NaOH. In this way, the substrate-to-protein ratio was maintained at a 10-fold molar excess, with the concentrations of *C*Cld and NaClO in the final sample being 0.25 and 2.5 mM, respectively. The samples containing chlorite were prepared in duplicate by mixing 50 μL of a solution containing *C*Cld at a concentration of 0.2 mM with 50 μL of a 60 mM sodium chlorite (NaClO_2_) solution prepared in Milli-Q water. In this case, the substrate-to-protein ratio was set at a 300-fold molar excess, with the concentrations of *C*Cld and NaClO_2_ in the final sample being 0.1 and 30 mM, respectively. Next, additional samples were prepared with a conventional “manual freezing” method. More specifically, to freeze the sample in the shortest time possible, the solution containing *C*Cld was first poured at the bottom of a quartz EPR tube, and then an appropriate volume of NaClO solution was added to the sample, quickly mixed, and immediately flash frozen in liquid N_2_ within 10 s. The same procedure was followed to prepare the sample in the presence of serotonin, which was added in a 50-fold molar excess before initiating the reaction cycle with NaClO.

Continuous wave (CW) EPR spectra were recorded with an X-band ELEXSYS E580 spectrometer (Bruker BioSpin GmbH) operating at a microwave frequency of ∼9.4 GHz and equipped with a standard TE102 cavity and a liquid He cryostat (Oxford Inc.). Measurements were performed at 10 K, under nonsaturating conditions, with a modulation frequency of 100 kHz and a modulation amplitude in the range of 0.2 to 1 mT according to the different experiments. For the determination of the EPR parameters, simulations were performed with the EasySpin toolkit (v. 6.0.0-dev.41) implemented in Matlab.^38^

### Chlorite Degradation Activity

Enzyme-mediated chlorite degradation was measured polarographically following the release of O_2_ by using a Clark-type oxygen electrode (Oxygraph Plus; Hansatech Instruments, Norfolk, UK). Reactions were monitored at 30 °C using a connected water bath. The electrode was calibrated by equilibrating to 100% O_2_ saturation by bubbling with air and to 0% O_2_ saturation by flushing with N_2_ until stable plateaus were reached to derive an offset and calibration factor. Reactions were performed in O_2_-free 50 mM phosphate–citrate buffer (pH 5.0), 50 mM phosphate buffer (pH 7.0), or 50 mM phosphate–borate buffer (pH 9.0), respectively. The substrate was added to final concentrations ranging from 20 to 1000 μM NaClO_2_. Concentrations of the chlorite stock solutions were determined using the molar extinction coefficient at 260 nm of 154 M^–1^ cm^–1^.^5,35^ Reactions were studied in the absence and presence of 100 μM serotonin or 1 mM methionine. The reaction was started by adding 20 nM wild-type *C*Cld or the variants Q74V or Q74E. Molecular oxygen production rates (μM O_2_ s^–1^) were determined from the initial linear time traces and plotted against chlorite concentrations.

### Stopped-Flow Spectroscopy

Pre-steady-state kinetic experiments were carried out with SX-18MV or Pi-star from Applied Photophysics using either a diode array detector or a monochromator and photomultiplier detector. Both conventional and sequential measurements were performed. The optical quartz cell had a path length of 10 mm and a volume of 20 μL. The fastest time for mixing was 0.68 ms. Conditions were as follows: 1.5 μM *C*Cld (final concentration) in 50 mM buffer (citrate–phosphate buffer (pH 5.0–6.0), phosphate buffer (pH 6.0–9.0), or borate–phosphate buffer (pH 9.0–10.0)) at room temperature. The conventional stopped-flow mode was used to (i) follow the reaction of ferric proteins with 500 μM chlorite in the absence or presence of serotonin and to (ii) follow Compound I formation by mixing the ferric proteins with 10–1000 μM hypochlorite.

To study the reactivity of Compound I, the sequential-mixing stopped-flow mode was used. In detail, 3 μM ferric *C*Cld in 50 mM citrate–phosphate buffer (pH 5.0) or phosphate buffer (pH 7.0) was mixed with a 10-fold excess of hypochlorite in 1 mM NaOH. For the determination of the concentration of the hypochlorite stock solutions, the molar extinction coefficient of 350 M^–1^ cm^–1^ at 292 nm was used.^5,36^ After a dead time of 75 ms, Compound I was mixed with varying concentrations of iodide, serotonin, chlorite, or hypochlorite. To trap Compound II/Compound I* (see below), the dead time was prolonged to 150 ms before mixing with serotonin.

For the determination of the impact of pH on Compound I stability, this redox intermediate was formed as described above and then, by using the pH-jump technique, was mixed with 100 mM buffers at selected pH values (citrate–phosphate buffer, pH 5–6; phosphate buffer, pH 6–9; borate–phosphate buffer, pH 9–10).

### MNP Assay

To evaluate (oxidative) modifications during the reaction of wild-type *C*Cld with ClO_2_^–^, spin trapping experiments were performed using MNP (2-methyl-2-nitrosopropane). MNP (5 mg) was dissolved in 100 μL RO-H_2_O and heated up to 60 °C in the dark. In the assay, 30 μM *C*Cld in 50 mM phosphate buffer (pH 7.0) in the absence and presence of 1 mM serotonin was used. The reaction was started by the addition of chlorite (0, 10, and 100 mM). The final MNP concentration was 5 mg mL^–1^.

The proteins were S-alkylated with iodoacetamide and digested with trypsin (Promega). The digested samples were loaded on a nanoEase C18 column (nanoEase M/Z HSS T3 Column, 100 Å, 1.8 μm, 300 μm × 150 mm, Waters) using 0.1% formic acid as the aqueous solvent. A gradient from 3.5% B (B: 80% acetonitrile, 20% A) to 40% B in 30 min was applied followed by a 5 min gradient from 40% B to 95% B that facilitates the elution of large peptides at a flow rate of 6 μL/min. Detection was performed with an ion-trap mass spectrometer (amaZon speed ETD, Bruker) equipped with the standard ESI source in positive ion, DDA mode (*i.e*., switching to MSMS mode for eluting peaks). Mass spectrometric scans were recorded (range: 150–2200 Da), and the eight highest peaks were selected for fragmentation. Instrument calibration was performed using an ESI calibration mixture (Agilent). The files were analyzed by manually searching for modified tyrosines (nitrotyrosine +44.98 Da).

### Spectroelectrochemistry

All experiments were conducted in a homemade OTTLE (optical transparent thin-layer spectroelectrochemical) cell as described recently.^11^ In detail, the three-electrode configuration consisted of a gold minigrid working electrode (Buckbee-Mears, Chicago, IL), a homemade Ag/AgCl/KCl_sat_ microreference electrode separated from the working solution by a Vycor set, and a platinum wire as the counter electrode. The reference electrode was calibrated against a saturated calomel (HgCl) electrode before each set of measurements. All potentials are referenced to the SHE (standard hydrogen electrode). Potentials were applied across the OTTLE cell with an Amel model 2053 potentiostat/galvanostat. A constant temperature was maintained by a circulating water bath, and the OTTLE cell temperature was monitored with a Cu-costan microthermocouple. UV–vis spectra were recorded using a Varian Cary C50 spectrophotometer. The OTTLE cell was flushed with argon gas to establish an oxygen-free environment in the cell. Conditions were as follows: 20 μM wild-type *C*Cld or variants in 100 mM potassium phosphate buffer (pH 7.0) plus 100 mM NaCl. Additionally, 220 μM methyl viologen and 2 μM lumiflavine-3-acetate, methylene blue, phenazine methosulfate, and indigo disulfonate were used as mediators.

## Results

### Electronic Configuration of Ferric Wild-Type *C*Cld and Variants Q74V and Q74E

All purified proteins showed a dimeric state, high purity (>90%), and an average *Reinheitszahl* (*A*_Soret_/*A*_280_) of 2.2, which demonstrates almost 100% heme occupancy (data not shown). UV–vis spectroscopic measurements at acidic and neutral pH demonstrate that the three heme proteins are in the ferric high-spin state, characterized by a Soret maximum at 406/408 nm; Q-bands at 502, 535, and 575 nm; and a charge transfer (CT)-band at 635 nm.^25−27^ Addition of a 1000-fold excess of serotonin, which in the following experiments is used as a one-electron donor of *C*Cld redox intermediates, had no impact on the electronic configuration of these iron-proteins as demonstrated by both UV–vis and ECD in the respective heme absorption area as well as by EPR spectroscopies (Figure S1 and Figure 6A,B). As serotonin exhibits strong absorbance maxima at 276 and 298 nm,^39^ this area is not shown in the UV–vis spectra.

### Reactions of Ferric *C*Cld and Variants with Chlorite at Acidic and Neutral pH Regimes Allow Trapping of Compound I

**Figure 2:**
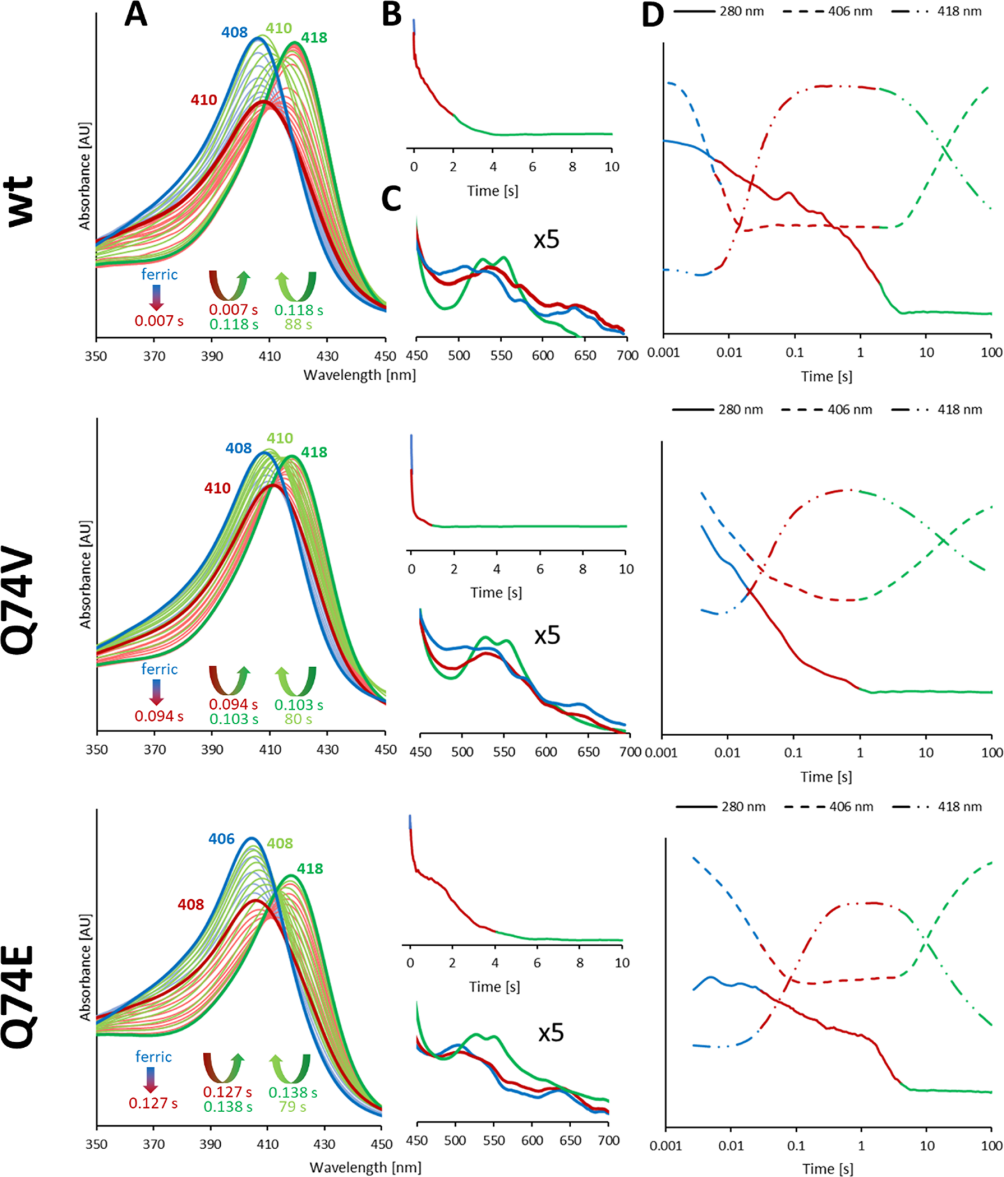
Reaction of wild-type *C*Cld and the variants Q74V and Q74E with ClO_2_^–^ at pH 7.0. Reactions were followed by conventional stopped-flow spectroscopy. Final concentrations: 1.5 μM enzyme, 500 μM chlorite. (A, C) Interconversion of redox intermediates during the reaction. The spectrum of the ferric protein and the spectral changes in the first fast phase of the reaction (*i.e.*, Compound I formation, red bold spectrum) are shown in blue. The second phase, *i*.*e*., formation of Compound II/Compound I*, is shown in red. The resulting species that dominates during chlorite degradation is shown in bold green. Spectral intermediates representing the slow final conversion back to the ferric resting state are displayed in green. (B) Chlorite degradation monitored by loss of absorbance at 280 nm. Color code corresponds to that of (A). (D) Time traces reflecting chlorite degradation (280 nm, solid line), formation of Compound I and resting state (406 nm, dashed line), as well as formation and conversion of Compound II/Compound I* (418 nm, dash-dotted line). The *x* axis is shown in logarithmic scale to have a better overview on the whole reaction.

To investigate the influence of the flexibility of R127 on the kinetics of the interconversion of the redox states of chlorite dismutase mediated by chlorite, conventional UV–vis stopped-flow spectroscopy was performed. Ferric wild-type *C*Cld, Q74V, or Q74E was mixed with a 330-fold excess of chlorite (1.5 μM enzyme, 500 μM chlorite), which guaranteed full degradation of chlorite and only a minor influence due to inactivation reactions of the enzymes over the whole pH range. Typically, a significantly prolonged phase of chlorite decomposition was observed with increasing pH. Interestingly, differences were observed in the time spans of chlorite degradation (monitored spectrophotometrically at 280 nm) by the three proteins following the hierarchy Q74V < wild-type *C*Cld < Q74E. At pH 5.0, *i.e*., the optimum pH of *C*Cld activity, these differences were relatively small [0.14 s (Q74V), 0.17 s (wild-type *C*Cld), and 0.58 s (Q74E)] (Figure S2) compared to pH 7.0 [1.4 s (Q74V), 4.2 s (wild-type *C*Cld), and 5.8 s (Q74E)] (Figure 2) and pH 9.0 [16 s (Q74V), 58 s (wild-type *C*Cld), and 90 s (Q74E)] (Figure S3).

Figure 2 depicts the interconversion of the redox intermediates of wild-type *C*Cld, Q74V, and Q74E during chlorite degradation at pH 7.0. In wild-type *C*Cld, immediately after mixing of the ferric protein with chlorite, Soret band absorbance at 408 nm is lost, suggesting Compound I formation (absorbance maximum ∼410 nm). Within ∼7 ms, maximum hypochromicity is reached, immediately followed by a rapid shift of the Soret maximum to 418 nm (7–118 ms) that dominates until chlorite is fully degraded after ∼5800 ms (Figure 2). The spectral features (418, 528, and 551 nm) of this redox intermediate are reminiscent of an oxoiron(IV) species [*e.g*. Compound II or Compound I* in heme peroxidases, the latter representing an oxoiron(IV) species plus a remote protein radical]. Finally, this redox intermediate is slowly and directly converted to the resting state (isosbestic point at 413 nm).

Similar spectral transitions are observed with the variants Q74V and Q74E (Figure 2), but the kinetics of interconversion of Fe(III) → Compound I → oxoiron(IV) species (Soret band at 418 nm) exhibited differences. In the case of Q74V, the hypochromicity of the Soret absorbance is less pronounced as a result of either slower Compound I formation or faster formation of the oxoiron(IV) species (Soret band at 418 nm). In Q74E, both reactions appear delayed (Figure 2).

At pH 5.0, chlorite degradation is significantly faster. When the iron(III) resting state enzymes are mixed with chlorite, similar hypochromicities are observed in the initial fast phase (resulting red bold spectra in Figure S2, absorbance maximum at 408 nm), and the kinetics is comparable to that at pH 7.0. Similar to pH 7.0, the hypochromicity at pH 5.0 follows the order Q74V < wild-type *C*Cld < Q74E. Interestingly, the Compound II/Compound I* oxoiron(IV) species fails to fully form. The resulting species that dominates during chlorite degradation exhibits Soret maxima around 406 nm (green bold spectra in Figure S2), whereas Compound II-/Compound I*-typical Q-band maxima at 528 and 551 nm do not occur. Finally, this redox intermediate slowly and directly converts back to the resting state.

At pH 9.0, chlorite is still fully degraded, but the reaction is very slow. In both the wild-type protein and the two mutant proteins, the ferric state appears to convert directly to the oxoiron(IV) species (418 nm) (Figure S3, bold green spectra, peaks at 418, 528, and 551 nm), which is very slowly converted to the ferric resting state after chlorite degradation.

### RFQ-EPR Spectroscopy of Wild-Type *C*Cld with ClO_2_^–^ at pH 5

Compound I has been characterized in many heme systems,^40^ and it can be described as a oxoiron(IV) delocalized porphyrin π-radical, being therefore a suitable target to be investigated by electron paramagnetic resonance (EPR) spectroscopy. In addition to that, whereas the oxoiron(IV) species Compound II would be EPR silent, the remote amino acid radical responsible for the quenching of the porphyrin radical in the so-called Compound I* species can be in principle detected and identified by means of this technique. Hence, we decided to use a combined rapid freeze-quench (RFQ)-EPR spectroscopy method to trap the fast-evolving intermediates formed during the catalytic reaction of *C*Cld in the presence of ClO_2_^–^. Figure S4C,D shows the CW X-band EPR spectrum of wild-type *C*Cld at pH 5, whose reaction in the presence of a 300-fold excess of chlorite was freeze-quenched within ∼60 ms. The full spectrum, represented in Figure S4C, is dominated by a strong signal in the high field region, with well-resolved hyperfine splittings. A weak signal at around 115 mT (*g* ∼5.9) indicates the presence of a residual high-spin signal from unreacted *C*Cld.^26^ The magnification of the dominating signal (solid black) and its corresponding simulation (dashed red) are depicted in Figure S4D. The spectral shape closely resembles that of a chlorine dioxide radical (ClO_2_^•^), as reported in several studies where gaseous ClO_2_^•^ was trapped in inert matrices^41^ or adsorbed in zeolite materials.^42^ Additionally, in a recent study on the pentameric Cld from *Dechloromonas aromatica,*(43) evidences of the EPR spectral signature of chlorine dioxide were reported for the reaction with chlorite at both pH 5.2 and 9.0. The species characterized in this work presents axial *g* and *A* tensors with principal values of *g* = [2.002 2.011 2.017] and *A* = [212 −36 −21] (in MHz), respectively. These parameters are in good agreement with the ones of the same radical species reported so far^41,42^ and are supported by density functional theory (DFT) calculations (Table S1). It is noteworthy that no evidences of the typical features of a Compound I signal, or those of an amino-acid radical, were observed in the spectrum. As will be discussed later, the strong signal from ClO_2_^•^, which likely represents a byproduct, might hamper the observation of weaker underlying signals.

### Compound I Stability Significantly Decreases with Increasing pH

**Figure 3:**
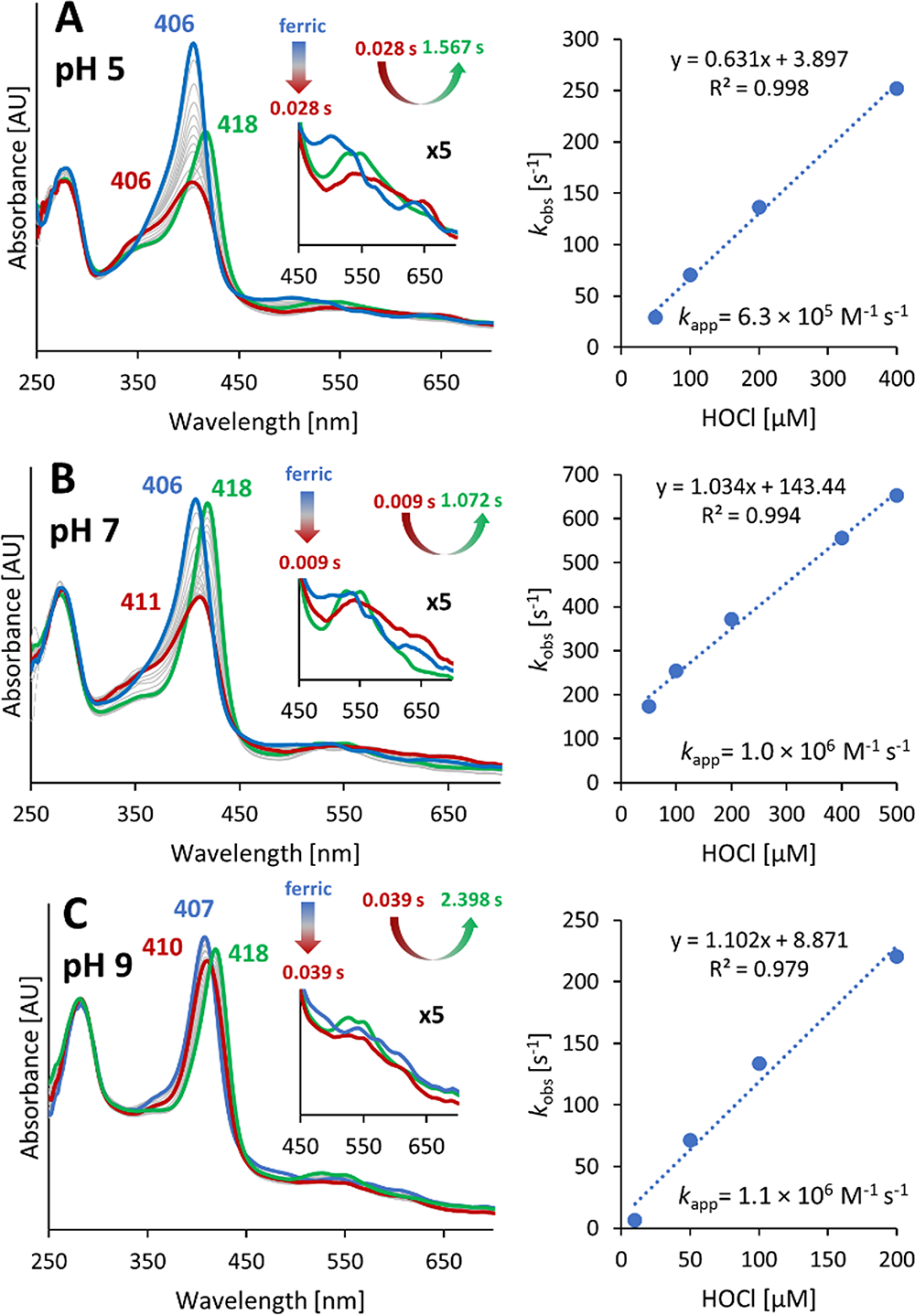
Reaction of ferric wild-type *C*Cld with hypochlorite at pH (A) 5.0, (B) 7.0, and (C) 9.0. The conversion of ferric wild-type *C*Cld to Compound I was mediated by mixing 1.5 μM enzyme with 200 μM hypochlorite in the conventional stopped-flow mode. Spectra of the ferric resting state, Compound I, and the oxoiron(IV) species are shown in bold blue, red, and green. The Q- and CT-band region (450–700 nm) is shown in fivefold magnification as insets. Right panels: plots of *k*_obs_*versus* hypochlorite concentration. The rate constant *k*_app_ of Compound I formation was obtained from the slope of the linear regression (right).

The previous experiments have shown that Compound I can be trapped at the beginning of the reaction between ferric *C*Cld and chlorite in the acidic and neutral pH range and that chlorine dioxide radicals (ClO_2_^•^) are formed, probably representing an off-pathway product through heme degradation. To probe the influence of both pH and flexibility of arginine on the rate of Compound I formation and its stability, the reactions of ferric *C*Cld and variants with hypochlorite were followed via conventional and sequential stopped-flow spectroscopy. Hypochlorous acid is known to convert heme proteins including peroxidases, catalases, and Clds to Compound I.^4,5,25,34,44^Figure 3 depicts the kinetics and spectral conversion of the reaction between wild-type *C*Cld and HOCl at pH 5.0, 7.0, and 9.0. At pH 5.0, this fast redox reaction (*k*_app_ = 6.3 × 10^5^ M^–1^ s^–1^) is accompanied by a ∼ 50% hypochromicity in the Soret region and the formation of a band at ∼650 nm (bold red spectrum in Figure 3A). At pH 5.0, Compound I of *C*Cld is relatively stable despite the fact that an excess of HOCl is present in the reaction mixture. This fact suggests that (at least in this experimental setup) reaction 2 (*i.e*., rebinding and reaction between hypochlorite and Compound I) does not occur. Finally, the spectral transitions suggest the (slow) conversion of Compound I to an oxoiron(IV) species with bands at 418, 528, and 550 nm, which might represent Compound I*.

At pH 7.0, a similar interconversion of *C*Cld redox intermediates occur; however, the formation of Compound I* is more pronounced compared to pH 5 (Figure 3B). Because the rate of Compound I formation is similar to that at pH 5.0, this clearly suggests a higher instability of Compound I and an increased rate of interconversion of Compound I to Compound I*. This is even more pronounced for the reaction at pH 9.0, where Compound I formation is already difficult to trap despite the fact that the rate of its formation is similar to that at pH 5.0 or 7.0 (Figure 3C). Both variants, Q74V and Q74E, show wild-type-like kinetics of Compound I formation in the entire pH range investigated (Figures S5 and S6).

**Figure 4:**
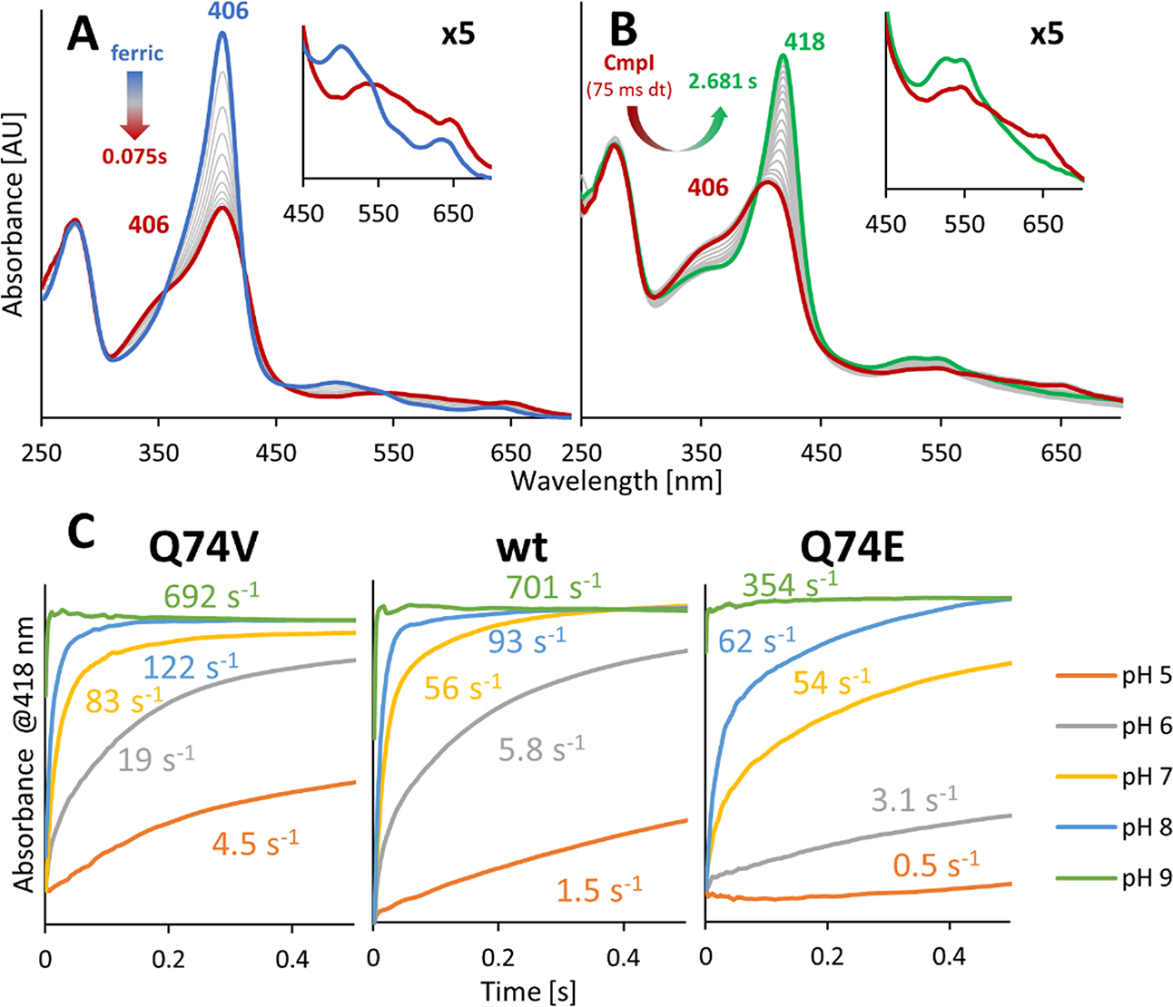
Impact of pH on the stability of Compound I of wild-type *C*Cld and the variants Q74V and Q74E. (A) Compound I formation upon mixing of 3 μM wild-type *C*Cld with 30 μM hypochlorite at pH 5.0. The spectrum of the ferric resting state and the formed Compound I is shown in blue and red, respectively. (B) After 75 ms delay time, Compound I was mixed with the buffer, pH 6.0, mediating the conversion of Compound I to Compound I* at this pH value. Spectra in the insets (in A and B) are fivefold magnified for better visualization of the Q- and CT-band region. (C) Time traces of the shift of Compound I to Compound I* of wild-type *C*Cld and the variants Q74V and Q74E followed at 418 nm. Values next to time traces represent the *k*_obs_ values for the decay of the respective Compound I.

Next, we studied the pH dependence of the stability of Compound I formed by HOCl in more detail by using the pH-jump technique. We first mixed ferric wild-type *C*Cld with a 10-fold excess of hypochlorite at pH 5 (Figure 4A), and after a delay time of 75 ms (full Soret hypochromicity), the resulting Compound I (red bold spectrum) was mixed with the buffer at selected pH values. Figure 4B depicts the decay of Compound I to Compound I* (green bold spectrum). As Figure 4C demonstrates, the stability of wild-type *C*Cld Compound I significantly decreases with increasing pH, with *k*_obs_ values of decay ranging from 1.5 s^–1^ (pH 5.0) to 701 s^–1^ (pH 9.0) in wild-type *C*Cld (Figure 4C). Importantly, the flexibility of R127 has an impact on Compound I stability following the hierarchy Q74E > wild-type *C*Cld > Q74V, the latter showing the least stability of Compound I between pH 5 and 8 (Figure 4C).

Because Figure 4 suggests differences in the redox properties between wild-type *C*Cld and the variants Q74V and Q74E, we decided to investigate the standard reduction potential of the couple Fe(III)/Fe(II) spectroelectrochemically. The representative families of spectra of ferric wild-type *C*Cld and the two variants at different applied potentials are very similar to those of pentameric and dimeric Clds investigated recently.^11,33^ Dimeric *C*Cld is directly reduced to its ferrous form with absorption maxima at 435 and 556 nm (not shown). The calculated midpoint potential of the Fe(III)/Fe(II) couple, determined from the corresponding Nernst plot, was calculated to be −95 ± 1.9 and −101 ± 2.1 mV for wild-type *C*Cld and Q74V, respectively. Arresting R127 in a salt bridge (Q74E) lowers *E*° (−112 ± 2.4 mV) according to the electrostatic stabilization of the ferric form.

### RFQ-EPR Spectroscopy of Wild-Type *C*Cld with ClO^–^ at pH 5

When the reaction of 0.25 mM wild-type *C*Cld with a 10-fold excess of ClO^–^ (2.5 mM) at pH 5.0 is freeze-quenched within ∼60 ms, a sharp featureless signal appears in the EPR spectrum around *g* ∼2.0 (Figure S7). Although Compound I would theoretically show a component at a similar field position,^17,^^45−48^ other factors suggest that the detected species may represent an amino-acid radical instead. On the one hand, the EPR spectra of Compound I described in previous studies of chlorite dismutases^43,48^ or other heme enzymes^17,45−47^ present some differences in terms of lineshape and broadening. Second, this signal is easily saturated at very low temperatures (2.5–5 K), and it can be observed up to 80 K (data not shown), which in fact excludes the possibility that it represents a Compound I species.^49^ Hence, this narrow line can be assigned to an unknown amino-acid radical, which would indicate the formation of Compound I*, the decay product of Compound I, in the absence of electron donors. The failure to observe an EPR signal due to Compound I despite the fact that the time range corresponds to its maximum formation according to stopped-flow experiments performed with the same substrate-to-protein ratio (Figure 4A) is undoubtedly due to the larger concentrations needed for the EPR experiment because the formation rate depends on the protein and substrate concentration. It has to be noted that when the sample is prepared with a “conventional” flash-freezing in liquid N_2_ (quenching time of ∼10 s), the same EPR signal is still visible, confirming the slow decay of the oxoiron(IV) species already observed in the UV–vis measurements. Figure S8 depicts a magnification of the high-field region in which the RFQ and the flash-frozen samples are compared. The two spectra only show a minor variation in lineshape, which may be due to small differences in the unresolved anisotropy of the EPR tensors or to the presence of a composite signal where the ratio of the individual components varies over time, which would also agree with the spin trapping results discussed later.

### Compound I of *C*Cld Reacts with Two- and One-Electron Donors but Not with HOCl

Next, we tested whether *C*Cld Compound I formed with HOCl is reactive toward one- or two-electron donors. The sequential-mixing stopped-flow technique was used; *i.e.*, first, Compound I was formed by mixing wild-type ferric *C*Cld (3 μM) with a 10-fold excess of hypochlorite at pH 5.0, and then, after a delay time of 75 ms, potential electron donors were added to the fully established Compound I. Importantly, further addition of HOCl did not promote the conversion of Compound I to the oxoiron(IV) species (not shown), suggesting that reaction 2 does not occur at these experimental conditions. By contrast, iodide efficiently reduced *C*Cld Compound I directly to the ferric resting state (9.4 × 10^5^ M^–1^ s^–1^) at pH 5 (Figure S9).

Next, we probed whether chlorite is able to act as a one-electron donor for *C*Cld Compound I and Compound II, as previously demonstrated with heme peroxidases.^4,5^Figure S10 shows the reaction between wild-type *C*Cld Compound I with chlorite at pH 5.0 and 7.0. At pH 5.0, 100 μM chlorite efficiently mediates the conversion of Compound I to the ferric state in a biphasic reaction, suggesting the reaction sequence Compound I → Compound II → ferric state (∼2.4 × 10^5^ and 2.0 × 10^4^ M^–1^ s^–1^) (Figure S10). At pH 5.0, Compound II fails to fully form during chlorite degradation (*i.e.*, 150 ms) (Figure S10). Only after complete chlorite degradation does the species with the Soret maximum at 418 nm become more pronounced.

At pH 7.0, 100 μM chlorite mediates the conversion of *C*Cld Compound I to the 418 nm species in a biphasic reaction with *k*_app_ values being ∼2.5 × 10^5^ and 8.8 × 10^4^ M^–1^ s^–1^, respectively (Figure S10). These data clearly show that Compound I can oxidize chlorite to chlorine dioxide and that the oxoferryl species (Compound II and/or Compound I*) also catalyze this reaction. At pH 9.0, the conversion of Compound I to the 418 nm species does not depend on the chlorite concentration owing to the rapid decay of Compound I to Compound I*.

**Figure 5:**
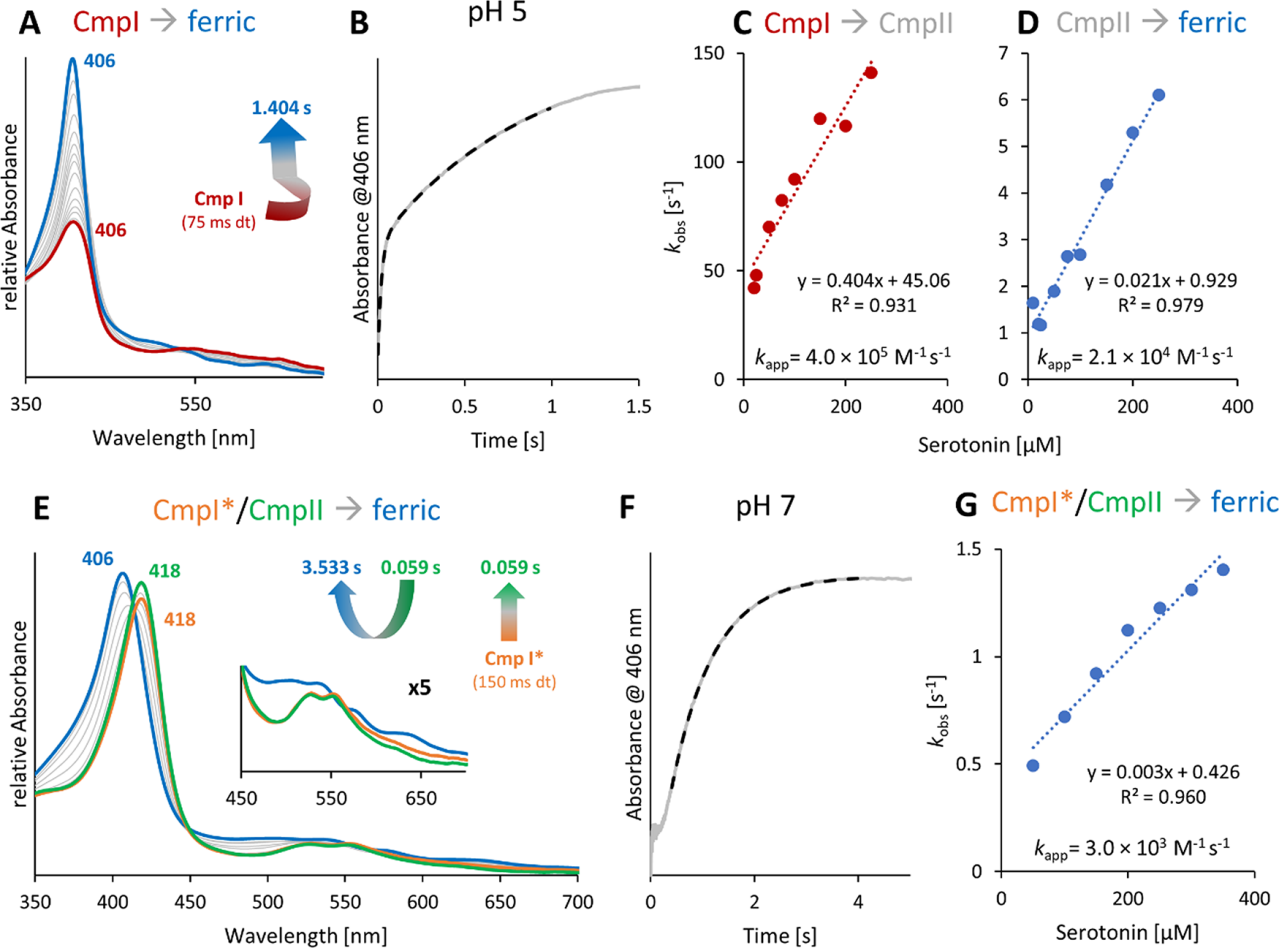
Reaction of Compound I and Compound I* with serotonin. (A) Biphasic reaction between Compound I (red spectrum), formed by mixing 1.5 μM wild-type ferric *C*Cld with a 10-fold excess of hypochlorite, and serotonin followed by the sequential-mixing stopped-flow technique at pH 5.0. Delay time: 75 ms. The resulting ferric resting state is shown in blue. (B) Typical time trace at 406 nm and double exponential fit, shown as solid gray and dashed black lines. (C, D) Corresponding linear plots of *k*_obs_*versus* serotonin concentration of the first and the second phase of the reaction. (E) Reaction of Compound I* (orange spectrum) with serotonin. Compound I* is the decay product of Compound I. At pH 7.0, Compound I is unstable and, in the absence of electron donors, rapidly decays to Compound I* within 120 ms. After a delay time of 150 ms, formed Compound I* (orange spectrum) was mixed with serotonin at pH 7.0. In the initial phase, Soret absorbance at 418 nm is increased (until 0.059 s, green spectrum), followed by direct monophasic conversion to the ferric state. (F) Representative time trace at 406 nm (solid gray line) and single exponential fit (dashed black line). (G) Corresponding linear plot of *k*_obs_*versus* serotonin concentration.

**Figure 6:**
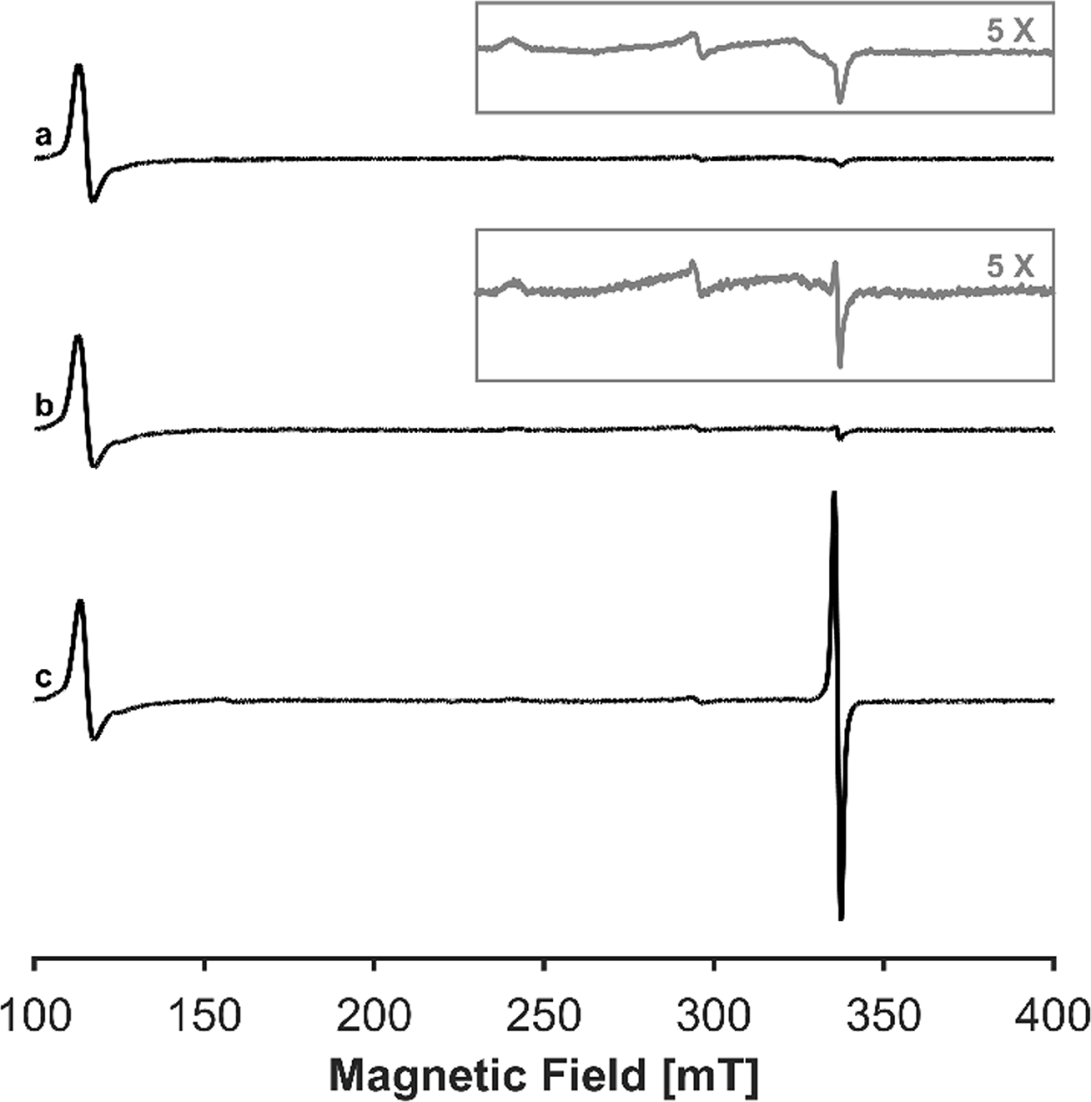
CW X-band EPR spectra of 0.2 mM wild-type *C*Cld in the (A) presence of serotonin alone and reacting with 2 mM hypochlorite at pH 5.0 in the (B) presence or (C) absence of 10 mM serotonin. The samples were prepared with a “conventional” liquid N_2_ flash-freezing method (quenching time < 10 s). The spectra were recorded at 10 K, with an MW power of 1 mW and a modulation amplitude of 1 mT. Intensities are normalized at 115 mT (*g*^eff^ ∼6 of high-spin component), and a 5× magnification of the high-field region is depicted in the insets for spectra (A) and (B).

Furthermore, we tested serotonin, an efficient one-electron donor for Compound I and Compound II of heme peroxidases.^39^ Serotonin reduced *C*Cld Compound I in a biphasic reaction (Figure 5B). Here, it is important to note that the presence of serotonin had no impact on the electronic signature of the heme iron (Figure S1 and Figure 6), suggesting that electron delivery would be limited to the heme periphery. The occurrence of a biphasic reaction in presence of serotonin indicates that the conversion of Compound I follows the sequence Compound I → oxoiron(IV) → Fe(III), where both steps exhibit a clear concentration dependency. At pH 5.0, the apparent second-order rate constants, *k*_app_’s, are calculated to be 4.0 × 10^5^ and 2.1 × 10^4^ M^–1^ s^–1^, respectively (Figure 5C,D). At pH 7.0, the *k*_app_ of Compound I reduction slightly increases (9.9 × 10^5^ M^–1^ s^–1^), whereas that of Compound II reduction slightly decreases (1.1 × 10^4^ M^–1^ s^–1^) (not shown).

Finally, we investigated whether serotonin is also able to react with Compound I*, the decay product of Compound I, in the absence of an external electron donor (Figure 4). Figure 5E depicts the reaction between serotonin and Compound I* at pH 7.0. In detail, after the HOCl-mediated Compound I formation at pH 7.0 and a delay time of 150 ms, fully formed Compound I* (418, 528, and 550 nm; orange spectrum) was mixed with serotonin (Figure 5E). The time trace at 406 nm (Figure 5F) shows an initial lag phase, which might reflect cycling due to excess of hypochlorite and/or formation of Compound II that exhibits the same UV–vis spectral properties as Compound I*. The final monophasic reaction, *i.e*., formation of the ferric state, shows a clear concentration dependency (*k*_app_ = 3.0 × 10^4^ M^–1^ s^–1^, Figure 5F,G), suggesting the occurrence of the following reaction, which could recover ferric *C*Cld while keeping the oxidized amino acid in the protein.

### EPR Spectroscopy of Wild-Type *C*Cld with ClO^–^ at pH 5 in the Absence or Presence of Serotonin

The ability of serotonin to prevent *C*Cld from undergoing a nonproductive pathway and forming Compound I* is confirmed by EPR spectroscopy findings. Figure 6 shows the EPR spectra of 0.2 mM *C*Cld in the presence of a 50-fold molar excess of serotonin (Figure 6A) as well as *C*Cld reacting with a 10-fold molar excess of hypochlorite (2 mM) in the presence (Figure 6B) or absence (Figure 6C) of serotonin at pH 5.0. The addition of serotonin alone has no effect on the spectrum of resting state *C*Cld, whose features have been previously reported.^26^ Interestingly, when serotonin is added to the protein sample prior to the addition of hypochlorite, the intensity of the protein radical signal described earlier (Figure S7 and Figure 6C) is substantially reduced (Figure 6B), indicating that serotonin is indeed able to act as a one-electron reductant of Compound I and Compound II as well as Compound I*.

### Serotonin Improves the Efficiency of Chlorite Degradation

**Figure 7:**
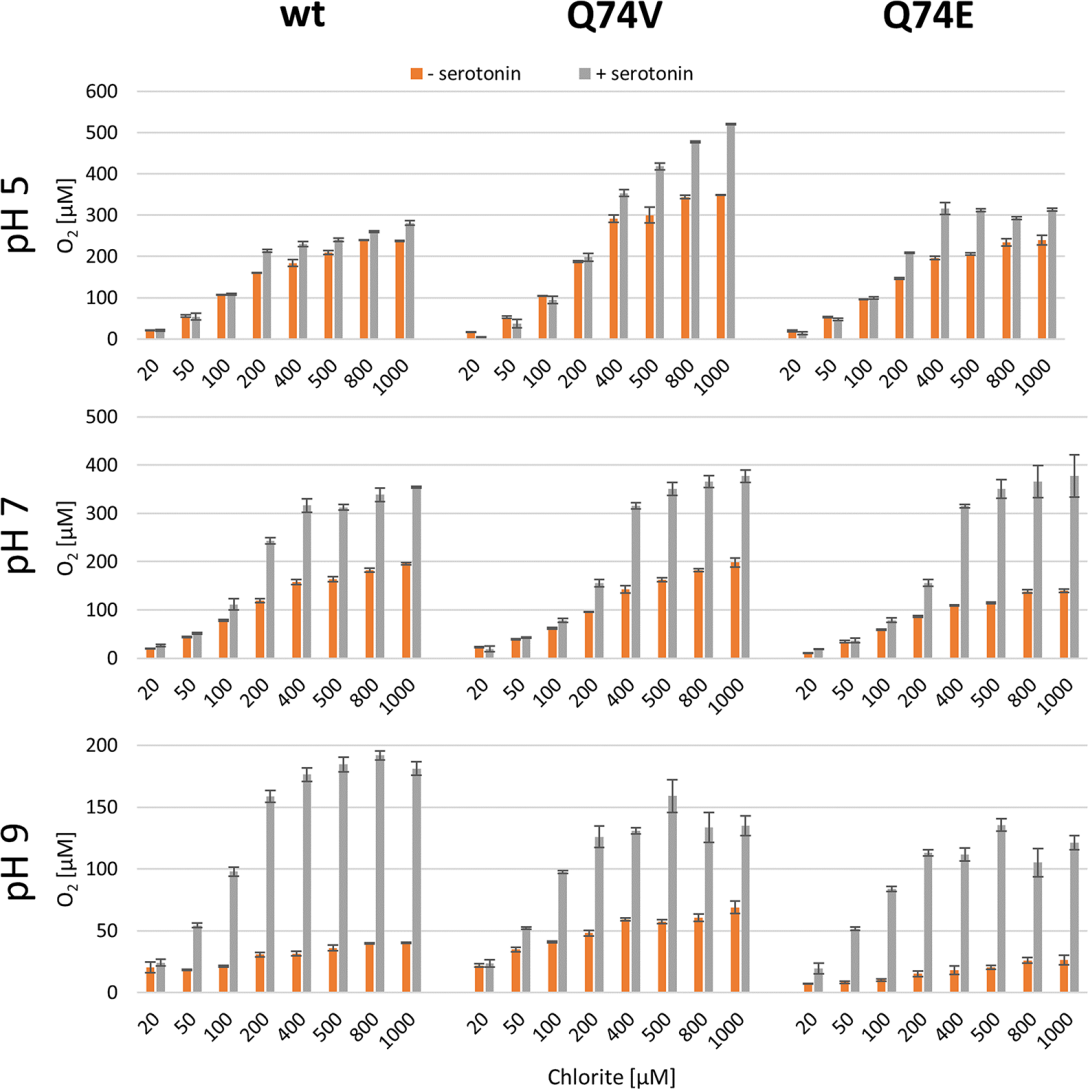
Influence of serotonin on O_2_ production of wild-type *C*Cld and the variants Q74V and Q74E at pH 5.0, 7.0, and 9.0. Total product (*i.e*., O_2_) formation is indicated by gray and orange bars for measurements in the presence (100 μM) or absence of serotonin. Conditions: chlorite concentration: 20–1000 μM, enzyme concentration: 20 nM. Measurements were done in triplicate.

Because serotonin can act as an electron donor for Compound I, Compound II, and Compound I*, we probed its impact on chlorite degradation (20–1000 μM) and dioxygen formation by wild-type *C*Cld (20 nM) and the variants Q74V and Q74E. Chlorite degradation was followed polarographically by using a Clark-type electrode. Figure 7 clearly depicts that all three proteins are prone to inhibition as reflected by the deviation from the stoichiometric ratio of 1:1 between chlorite and O_2_. This deviation is more pronounced at higher pH values and most obvious for Q74E (Figure 7). Most interestingly, the presence of 100 μM serotonin protects wild-type *C*Cld and the variants from inhibition to some extent. This protective effect is relatively small at the pH optimum but becomes more relevant with increasing pH (Figure 7). By contrast, the impact of methionine on rescuing *C*Cld from inactivation is marginal (data not shown), although this compound is known to trap HOCl.^51^

It is important to note that the beneficial effect of serotonin on chlorite degradation is highest at a twofold excess of serotonin compared to chlorite but decreases when a larger excess is used. Under these conditions, serotonin has no significant impact on the kinetic parameters *K*_M_ and *k*_cat_ and in consequence on the *k*_cat_/*K*_M_ values published recently (not shown).^26,27^ At higher concentrations (serotonin/chlorite ratios >10), the chlorite degradation activity decreases (Figure S11), suggesting that (i) serotonin competes with distinct reaction steps in enzyme turnover and (ii) its binding in the heme periphery does not interfere with chlorite binding.

Finally, we probed the impact of 100 μM serotonin on the spectral transitions induced by the addition of chlorite to wild-type *C*Cld and the variants Q74V and Q74E at pH 5.0 (Figure S12), pH 7.0 (Figures S13), and pH 9.0 (Figure S14), respectively. Compared to the reactions without serotonin (see above), very similar spectral transitions were observed. The phase of chlorite decomposition is not significantly influenced by the addition of serotonin, as the time required for complete chlorite decomposition does not significantly change. Similar to the reactions without serotonin, the efficiency of chlorite degradation followed the hierarchy Q74V > wild-type *C*Cld > Q74E. For all three enzymes, the oxoiron(IV) species converts back to the ferric resting state about 10 times faster in the presence of serotonin.

### Serotonin Suppresses Oxidative Damage of *C*Cld by Chlorite

As outlined above, *C*Cld Compound I is rapidly converted to Compound I* in the absence of external electron donors (Figure 5). This implies the formation of protein radicals, as also highlighted by EPR spectroscopy results. Because Compound I* can be rescued by serotonin to some extent (as the oxidative damage in the protein remains), we wondered whether this aromatic one-electron donor is able to dampen oxidative damage of this heme protein incubated with chlorite. To trap potential protein radicals, we used the spin trap 2-methyl-2-nitrosopropane (MNP) that is known to specifically attack and modify tyrosyl radicals, yielding 3-nitrotyrosine.^51^ The latter can be detected and analyzed by mass spectrometry.

**Figure 8:**
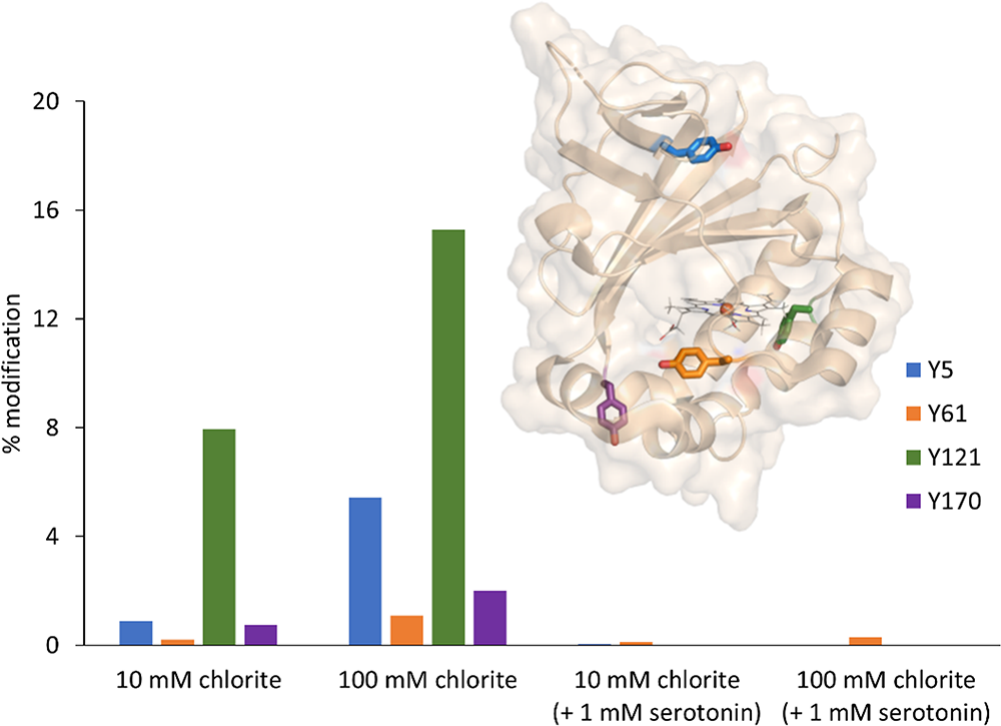
Serotonin suppresses tyrosyl radical formation in wild-type *C*Cld. Protein radical formation was analyzed by mass spectrometry using 2-methyl-2-nitrosopropane (MNP) as spin trap. MNP-assay measurements were done for two different substrate concentrations, 10 and 100 mM chlorite, and in the presence (1 mM) and absence of serotonin at pH 7.0. *C*Cld concentration: 30 μM. Modified tyrosine residues Y5 (blue), Y61 (orange), Y121 (green), and Y170 (violet) are shown in stick representation. To demonstrate their position within the protein, one monomer is shown in transparent surface and cartoon representation (light orange). All measurements were done in duplicate.

Chlorite dismutase from *Cyanothece* sp. PCC7425 has four tyrosine residues per monomer that could potentially be modified. We did not find modifications in the absence of chlorite (data not shown). In the presence of high chlorite concentrations (10 mM chlorite, *i.e*., 300-fold excess, or 100 mM chlorite, *i.e*., 3000-fold excess), all tyrosine residues show a modification to a certain degree. Residues Y5 and Y121 exhibit the highest level of modification with up to 16%, whereas the other two tyrosine residues, Y61 and Y170, are only slightly modified. The tyrosyl radical formation also shows a clear concentration dependence because the extent of modification increased at higher chlorite concentrations (Figure 8). Interestingly, only modest modifications were observed when serotonin was present in the reaction mixture.

## Discussion

It has been demonstrated with pentameric (clade 1) Cld from *Dechloromonas aromatica* (*Da*Cld) that chlorite is the sole source of dioxygen as determined by oxygen-18 labeling studies and that Cld uses chlorite neither for oxygen atom transfer to aliphatic or aromatic molecules nor in halogenation reactions.^48^ This distinguishes Clds from peroxidases and other heme enzymes. By contrast, when peroxyacetic acid was used as an alternative oxidant of *Da*Cld, oxidation and oxygen atom transfer reactions occurred and Compound I [oxoiron(IV) porphyryl radical] could be trapped by rapid-mixing UV–visible spectroscopy.^30^ Peroxyacetic acid (PAA) is an extremely strong oxidant with *E*°′[(CH_3_COOOH, H^+^/CH_3_COO^–^, H_2_O) = 1636 mV at pH 6.0],^52^ known to mediate the two-electron oxidation of heme proteins. Whereas reactions with hydrogen peroxide resulted in slow heme destruction,^30^ at acidic pH, heterolytic cleavage of the O–O bond of PAA yielded clean *Da*Cld Compound I.^30^ At alkaline pH, rapid formation of Compound I* [*i.e*., oxoiron(IV) protein radical] with an uncoupled protein-based radical verified by EPR was observed. The authors concluded that PAA mediates Compound I formation at all pH values and that radical migration is strongly promoted at alkaline pH regimes. Additionally, the authors proposed that (by analogy with PAA) the heterolytic Cl–O bond cleavage of chlorite to yield a oxoiron(IV) porphyrin cation radical is the most likely initial step in Cld catalysis, accompanied, therefore, by O–O bond formation from Compound I and hypochlorite.^30^ Recently, Geeraerts *et al.* demonstrated the formation and accumulation of Compound I in the reaction between *Da*Cld and bromite.^43^ Bromite was used as the surrogate substrate because the reaction between *Da*Cld and chlorite did not allow clear assignments of the relevant catalytic intermediates. Importantly, bromite decomposition was coupled with the evolution of O_2_.^43^

### Formation and Reactivity of Compound I of *C*Cld

Here, we have investigated the dimeric (clade 2) Cld from *Cyanothece* sp. PCC7425 (*C*Cld). We demonstrate that, similar to PAA, the surrogate oxidant hypochlorite is also able to mediate the two-electron oxidation of ferric Cld to Compound I. Hypochlorous acid is a strong two-electron oxidant [*E*°′ (HOCl, H^+^/Cl^–^, H_2_O) = 1280 mV at pH 7.0].^34^ At pH 5.0, *C*Cld mediates the heterolytic cleavage of the O–Cl bond of hypochlorite, yielding Compound I. The spectral signatures, *i.e*., 50% hypochromicity in the Soret region and formation of a band at ∼650 nm, clearly suggest the presence of an oxoiron(IV) porphyryl radical species. The apparent second-order rate constant of this reaction slightly increases with increasing pH; *i.e*., the rate at pH 9.0 is 75% higher than that at pH 5 (*i.e*., 6.3 × 10^5^ M^–1^ s^–1^) (Figure 3).

Similar to Compound I of heme peroxidases, *C*Cld Compound I is able react with two- and one-electron donors like iodide and serotonin and also with chlorite. With iodide, the reduction of *C*Cld Compound I was monophasic and directly converted this intermediate to ferric *C*Cld. By contrast, reduction by one-electron donors like serotonin and chlorite was biphasic, suggesting the reaction sequence Compound I → Compound II → ferric *C*Cld. One-electron oxidation of chlorite by the redox intermediates Compound I and Compound II of heme peroxidases produces chlorine dioxide, ClO_2_^•^.^5^ The standard reduction potential, *E*°′, of the couple (ClO_2_/ClO_2_^–^) has a value of 934 mV and is independent of pH above pH 2.0.^25,35^ The fact that *C*Cld Compound I and Compound II are also able to rapidly produce chlorine dioxide at rates of ∼10^5^ M^–1^ s^–1^ indicates that (i) the respective *E*°′ values of the redox couples Compound I/Compound II and Compound II/ferric *C*Cld must be >1000 mV and (ii) these reactions could contribute at least as side reactions in Cld turnover under certain reaction regimes, *e.g*., excess of chlorite. This is underlined by the fact that during chlorite degradation, an increase in absorbance at 360 nm has been observed,^25^ a wavelength at which chlorine dioxide exhibits its characteristic absorption maximum.^45^ In addition, the formation of chlorine dioxide in the Cld/chlorite system could by demonstrated by EPR with *C*Cld (present study) but also with pentameric *Da*Cld.^43^

### pH-Dependent Conversion of Compound I to Compound I*

Similar to clade 1 *Da*Cld Compound I formed by PAA, the stability of *C*Cld Compound I formed by hypochlorite significantly decreased with increasing pH. Upon combining sequential-mixing stopped-flow spectroscopy with the pH-jump technique, we were able to evaluate the kinetics of interconversion of Compound I to Compound I*, the latter having the porphyryl radical quenched by electrons spent by the protein matrix. Thus, Compound I* formation is accompanied by formation of amino acid radicals (reaction 5) and in consequence by oxidative damage of the protein matrix, which has been demonstrated by spin-trapping experiments using MNP combined with mass spectrometric analysis as well as by EPR.
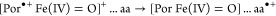
5

*C*Cld Compound I* exhibits spectral characteristics of an oxoiron(IV) intermediate with an uncoupled protein radical, reflected by absorbance maxima at 418, 528, and 550 nm. It is shown that the rate of this internal electron transfer from the protein to Compound I is almost 500 times higher at pH 9.0 compared to that at pH 5.0, suggesting the presence of proton-coupled electron transfer as recently described for *Da*Cld.^43^

Furthermore, we could demonstrate that the dynamics of the distal catalytic arginine R127 also impacts the kinetics of Compound I* formation, as shown by the comparison of wild-type *C*Cld with two recently designed variants.^26,27^ These three proteins differ in the distal heme cavity architecture having the catalytic arginine R127 (i) hydrogen-bonded to glutamine Q74 (wild-type *C*Cld), (ii) arrested in a salt bridge with a glutamate (Q74E), or (iii) being fully flexible (Q74V). The effect of this modulation of the noncovalent interactions of R127 and its impact on the dynamics of R127 have been demonstrated by (i) high-resolution crystal structures and molecular dynamic simulations of the respective proteins, (ii) the pH dependence of the electronic and spectroscopic signatures evaluated by UV–vis and EPR spectroscopies, and (iii) the impact of the flexibility of R127 on the conformational and thermal stability.^26,27^ Here, we could demonstrate that the dynamics of R127 does not affect the formation of Compound I mediated by hypochlorite but has an influence on Compound I stability following the hierarchy Q74E > wild-type *C*Cld > Q74V. Apparently, the decreased residence time of the positive guanidinium group in the vicinity of the heme iron in Q74E decreases the oxidation capacity of Compound I and in consequence increases the stability of Compound I. This is partly reflected by the more negative *E*°′ value for the redox couple Q74E compared to the other two proteins, as the molecular factors that determine *E*°′[Fe(III)/Fe(II)] also influence *E*°′ of the catalytically relevant Compound I/Fe(III) and Compound I/Compound II redox couples.^11,13−15,18,22^

Importantly, Compound I* formation occurs both in the system *C*Cld/hypochlorite and in the system *C*Cld/chlorite, and all tyrosines are modified in the presence of excess oxidants. This clearly suggests that (i) Compound I* is not directly involved in catalysis and (ii) the internal electron transfer from the protein to Compound I is unspecific in *C*Cld. By contrast, in DaCld, a specific electron transfer from Y118 to propionate 6 of the heme was postulated.^43^ In *C*Cld, Y121 shows the highest modification by MNP (Figure 8). Compared to the other tyrosines (Y5, Y170, Y61), Y121 exhibits both a relatively short distance to the heme iron (12.9 Å) and noncovalent interactions with E157 and R161.

In any case, the formation of Compound I* can be correlated with the fact that the Cld activity significantly decreases with increasing pH values. Interestingly, serotonin acts as a one-electron donor not only for *C*Cld Compound I and Compound II but also for Compound I*, thereby providing the possibility to get the enzyme out of this dead end and back to catalytically active intermediate(s). Here, it is important to mention that, in contrast to Compound I and Compound II reduction by serotonin to the ferric state, reduction of Compound I* by serotonin must leave oxidative damage in the protein matrix (reaction 6) because serotonin is not able to reduce the various remote protein radical sites.
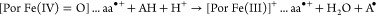
6

In addition, serotonin acts as an electron donor for Compound I, thereby dampening reaction 5. This is also corroborated by EPR spectroscopy, where the addition of a 50-fold molar excess of serotonin before starting the reaction with hypochlorite had a clear impact in the resulting spectrum. The signal attributed to the amino acid radical associated to Compound I* formation is greatly reduced in the presence of serotonin (Figure 6). In any case, serotonin partly prevents inhibition of *C*Cld, and this effect is most pronounced at higher pH values. This is an important finding for the use of chlorite dismutase in a wide variety of applications.^6^ Future studies will have to determine which cost-effective substitute for serotonin can be used as a single-electron donor in practice to improve the performance of this heme enzyme.

### Compound I Is the Catalytically Active Redox Intermediate during Chlorite Degradation

The question remains about the catalytically relevant redox intermediate in chlorite degradation. The provided stopped-flow data demonstrate that upon mixing of *C*Cld with chlorite, Compound I is the dominating redox intermediate at optimum pH. An oxoiron(IV) species with Soret maximum at 418 nm fails to fully form at pH 5.0. With increasing pH, the oxoiron(IV) species dominates the spectral signatures during chlorite degradation. In principle, both Compound II and Compound I* can contribute to these spectral features. As outlined above, the dominance of the oxoiron(IV) species during turnover at neutral and alkaline pH could have two causes: (i) the decrease in the oxidation capacity of chlorite and (ii) the increasing instability of Compound I at higher pH values. In any case, the present study clearly demonstrates that Compound I conversion to Compound I* (reaction 5) is an important side reaction of dimeric chlorite dismutases that plays an increasingly important role with rising pH values. Nevertheless, chlorite is also degraded at high pH values like pH 9.0, albeit very slowly and incompletely. This could be based on the fact that chlorite is able to reduce Compound I* according to reaction 6, thereby recovering the ferric state and making it possible for the enzyme to go through a few more cycles.

The fact that (unlike in stopped-flow spectroscopy) it was not possible to identify the EPR signature of *C*Cld Compound I in the presence of chlorite could be due to the working conditions of EPR spectroscopy, which require a much higher concentration of the reagents. This scaling up could promote off-pathway reactions (*i*.*e*., Compound I* formation) and induce a greater production of chlorine dioxide, whose strong spectral features can easily cover any weaker underlying signals (Figure S4). This would also explain why, with a similar substrate-to-protein ratio and same time range, the characteristic chlorine dioxide band at 360 nm is not visible in the stopped-flow experiments performed in this work. Our results are consistent with the recent work of Geeraerts *et al.*,^43^ who described the formation of chlorine dioxide during the reaction of the pentameric Cld from *Da*Cld with a high excess of chlorite. To our knowledge, this is the only reported EPR signal assigned to a ClO_2_^•^ radical in chlorite dismutase studies. However, it is important to note that similar spectra reported in previous works were given a completely different interpretation. In particular, in the seminal study of Lee *et al.*(48) on *Da*Cld, the EPR spectrum of the enzyme in the presence of chlorite was dominated by an intense signal whose features are remarkably similar to the ones we here unambiguously assign to chlorine dioxide. Hence, in the absence of simulation data and a zoomed image of this spectral area, the authors’ assignation to a tryptophyl radical is not convincing to us. More recently, the group of Püschmann *et al.*(53) proposed a brand new mechanism for chlorite dismutases on the basis of hyperquenched EPR spectroscopy results. The authors reported an EPR spectrum recorded <100 μs after the start of the reaction with chlorite and described the obtained signal as a triplet state resulting from two coupled amino-acid cation radicals. However, the spectrum shows a significant resemblance with that of chlorine dioxide, and the attempt of the authors to exclude this hypothesis by highlighting the absence of “satellite” signals is, in our opinion, undermined by poor signal-to-noise ratio.

If Compound I is the relevant catalytic redox intermediate in Cld catalysis, the two-electron oxidation of ferric Cld to Compound I must include heterolytic cleavage of chlorite to hypochlorite (reaction 1) that must stay in the reaction sphere and rearrange, rebind, and react with Compound I to produce O_2_ and Cl^–^ (reaction 2). The absence of any reaction between Compound I and HOCl as demonstrated in the stopped-flow experiments might, however, contradict an efficient rebound mechanism between Compound I and hypochlorite. An analogous experiment was performed with *Da*Cld and PAA followed by addition of hypochlorite. Similarly to the present study, no reaction of Compound I with hypochlorous acid was observed, and no O_2_ formation could be detected.^43^ However, in contrast to the natural substrate chlorite that reacts with the ferric enzyme to form the geminate pair Compound I/OCl^–^, exogenous hypochlorite may not arrange correctly with the oxoiron(IV) species or even has limited access to the active center, and Compound I decays to Compound I* before reaction with hypochlorite. In addition, as outlined by Geeraerts *et al*.,^43^ the absence of a catalytic base in Cld hinders the deprotonation of HOCl at neutral and acidic pH regimes, which is a precondition for O–O bond formation between the ferryl oxygen and hypochlorite. It has to be mentioned that reaction 2 was ruled out by DFT calculations because of an unfavorable high energy barrier, although the model used was very simple and does not reflect the realities of the heme pocket of a Cld.^54^

In any case, use of the stronger surrogate two-electron oxidant hypochlorite allowed the formation of pure Compound I in the neutral and alkaline pH regimes and in consequence the investigation of its reactivity and stability. Compound I of *C*Cld is shown to act as both two-electron (reaction 7) as well as one-electron acceptor (reaction 8), with AH representing serotonin. Upon one-electron reduction of Compound I, Compound II is formed (reaction 8) that further acts as a one-electron acceptor, thereby recovering the ferric resting state (reaction 9). Thus, both Compound I and Compound II generate radicals including chlorine dioxide, which is thus a byproduct of chlorite degradation by Cld.
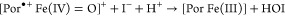
7
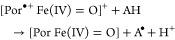
8
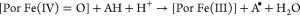
9

### New Insight into the *C*Cld Reaction Mechanism

Recently, we have demonstrated that the flexibility of the catalytic arginine in *C*Cld has a strong impact on the thermodynamics of binding of the angulate oxoanions nitrite and chlorite. At the optimum pH of *C*Cld (*i.e*., pH 5.5) and neutral pH, the *K*_D_ value for nitrite and the *K*_M_ value for chlorite are lowest when R127 is highly flexible and highest when R127 exhibits a salt bridge with E74.^27^ Immediately after substrate binding via O-ligation to Fe(III) and concomitant establishment of H-bonds between the two oxygen atoms of chlorite with R127 in the “in” conformation,^27^ the redox reaction takes place, and chlorite mediates the two-electron oxidation of ferric *C*Cld to Compound I according to reaction 1. Thermodynamically, this reaction is favored at optimum pH (*i.e*., pH 5.5); however, it becomes less favorable with increasing pH. During the oxidation of ferric *C*Cld to Compound I, the flexibility of R127 does not matter because it is already in the “in” conformation as a result of ligand binding even in Q74E.^27^ The pronounced H-bonding network between the *C*Cld-chlorite complex and R127^27^ as well as the potential role of the guanidinium group in heterolytic cleavage of chlorite and in H-bonding of the transient intermediate hypochlorite can also contribute to the fact that Compound I does not recombine when hypochlorite is added from outside to an already preformed Compound I.

The present study clearly demonstrated that the flexibility of R127 also impacts (i) the length in the time spans of chlorite degradation as well as (ii) the stability of Compound I. During the recombination reaction between Compound I and hypochlorite, the catalytic R127 must stay in the “in” conformation to keep the transient (oxidizing and chlorinating) intermediate hypochlorite in the reaction sphere. Recombination implies rotation of hypochlorite and rearrangements of hydrogen bonds to avoid escape of hypochlorite and oxidative damage of the protein.^50^ A reduced flexibility of R127 or even its elimination promotes (significant) decrease in activity^19−30^ and release of hypochlorite.^50^ In addition, the rate of Compound I to Compound I* conversion is increased with increased pH values, which additionally contributes significantly to the loss of activity above the optimum pH. Whether homolytic cleavage of chlorite and thus Compound II formation play a role in the alkaline range cannot be concluded from the available data, especially because Compound II and Compound I* exhibit very similar UV–vis spectra. In any case, the present study has demonstrated that serotonin is able to dampen the inhibition of Cld as a result of its ability to reduce oxoiron(IV) species including Compound I, Compound II, and Compound I*. This is an important finding for future practical applications of chlorite dismutases (*e.g.*, in bioremediation).^6^

## Supporting Information Available

Calibration of the rapid freeze-quench (RFQ) device from BioLogic; density functional theory (DFT); references (supporting materials and methods); comparison between the DFT-computed *g* and 35Cl hyperfine values for chlorine dioxide and the parameters used to simulate the EPR spectrum in Figure S4D (Table S1); impact of serotonin on the UV–vis and electronic circular dichroism spectral features of wild-type *C*Cld at pH 7.0 (Figure S1); reaction of wild-type *C*Cld and the variants Q74V and Q74E with ClO_2_^–^ at pH 5.0 and 9.0 (Figures S2 and S3); calibration of the rapid freeze-quench device from BioLogic and CW X-band EPR spectrum of wild-type *C*Cld in the presence of a 300-fold molar excess of chlorite (Figure S4); interconversion of spectral features of wild-type *C*Cld and the variants Q74V and Q74E mediated by hypochlorite at pH 5.0 (Figure S5); kinetics of Compound I formation of the *C*Cld variants Q74V and Q74E at pH 5.0, 7.0, and 9.0 (Figure S6); CW X-band EPR spectrum of wild-type *C*Cld in the presence of a 10-fold molar excess of hypochlorite at pH 5.0 (Figure S7); CW X-band EPR spectra of wild-type *C*Cld in the presence of a 10-fold molar excess of hypochlorite at pH 5.0 obtained by RFQ and N_2_ flash-freezing (Figure S8); reaction of wild-type *C*Cld Compound I with iodide at pH 5.0 (Figure S9); reaction of wild-type *C*Cld Compound I with chlorite at pH 5.0 and 7.0 (Figure S10); impact of serotonin on chlorite degradation and O_2_ production at pH 7.0 (Figure S11); and reaction of wild-type *C*Cld and the variants Q74V and Q74E with ClO_2_^–^ in the presence of serotonin at pH 5.0 (Figure S12), pH 7.0 (Figure S13), and pH 9 (Figure S14), respectively (PDF)

## Accession Codes

Cld from *Cyanothece* sp. PCC7425 (CCld), UniProt entry B8HNS6.

## Abbreviations

Cld: chlorite dismutase
*C*Cld: chlorite dismutase from *Cyanothece* sp. PCC7425
CcP: cytochrome *c* peroxidase
*Da*Cld: chlorite dismutase from *Dechloromonas aromatica*
*E*°′: standard reduction potential
ECD: electronic circular dichroism
EPR: electron paramagnetic resonance
HRP: horseradish peroxidase
LPO: lactoperoxidase
MNP: 2-methyl-2-nitrosopropane
MPO: myeloperoxidase
PAA: peracetic acid
RFQ: rapid freeze quench
