# Supplementary material for: Compound I Formation and Reactivity in Dimeric Chlorite Dismutase: Impact of pH and the Dynamics of the Catalytic Arginine
Source: Biochemistry. 2023 Jan 27;62(3):835–50. doi: 10.1021/acs.biochem.2c00696 (PMC9910045; doi:10.1021/acs.biochem.2c00696)
Supplement: Supplementary file 1 — bi2c00696_si_001.pdf [file bi2c00696_si_001.pdf]

Supporting Information

Compound I formation and reactivity in dimeric chlorite dismutase – Impact of pH and the dynamics of the catalytic arginine

Daniel Schmidt[1], Nikolaus Falb[1], Ilenia Serra[2], Marzia Bellei[3], Vera Pfanzagl[1], Stefan Hofbauer[1], Sabine Van Doorslaer[2] Gianantonio Battistuzzi[4], Paul G. Furtmüller[1] and Christian Obinger[1*]

[1]Institute of Biochemistry, Department of Chemistry, University of Natural Resources and Life Sciences, Vienna, Muthgasse 18, A-1190 Vienna, Austria

[2]BIMEF Laboratory, Department of Chemistry, University of Antwerp, Belgium

[3]Department of Life Sciences, University of Modena and Reggio Emilia, 41100 Modena, Italy

[4]Department of Chemistry and Geology, University of Modena and Reggio Emilia, 41100 Modena, Italy

*Corresponding author:

Christian Obinger: University of Natural Resources and Life Sciences, Department of Chemistry, Institute of Biochemistry, Vienna, Muthgasse 18, 1190 Vienna, Austria. Phone: +43-47654-77273, Fax: +43-1-47654-77250. E-mail: christian.obinger@boku.ac.at

Supporting Information – Table of Contents

Supporting Materials and Methods **S3**

Table S1: Comparison between the DFT-computed g and 35Cl hyperfine values for chlorine dioxide and the parameters used to simulate the EPR spectrum in Figure S4D **S7**

Figure S1: Impact of serotonin on the UV-vis- and electronic circular dichroism spectral features of wild-type *C*Cld at pH 7.0 **S8**

Figure S2 and S3: Reaction of wild-type *C*Cld and the variants Q74V and Q74E with ClO_2^- at pH 5.0 and pH 9.0 **S9**

Figure S4: Calibration of the rapid freeze-quench device from BioLogic and CW X-band EPR spectrum of wild-type *C*Cld in presence of a 300-fold molar excess of chlorite **S11**

Figure S5: Interconversion of spectral features of wild-type *C*Cld and the variants Q74V and Q74E mediated by hypochlorite at pH 5.0 **S12**

Figure S6: Kinetics of Compound I formation of the *C*Cld variants Q74V and Q74E at pH 5.0, 7.0 and 9.0 **S13**

Figure S7: CW X-band EPR spectrum of wild-type *C*Cld in presence of a 10-fold molar excess of hypochlorite at pH 5.0 **S14**

Figure S8: CW X-band EPR spectra of wild-type *C*Cld in presence of a 10-fold molar excess of hypochlorite at pH 5.0 obtained by RFQ and N_2 flash-freezing **S15**

Figure S9: Reaction of wild-type *C*Cld Compound I with iodide at pH 5.0 **S16**

Figure S10: Reaction of wild-type *C*Cld Compound I with chlorite at pH 5.0 and 7.0 **S17**

Figure S11: Impact of serotonin on chlorite degradation and O_2 production at pH 7.0 **S18**

Figure S12, S13 and S14: Reaction of wild-type *C*Cld and the variants Q74V and Q74E with ClO_2^- in the presence of serotonin at pH 5.0, pH 7.0 and pH 9 **S19**

Supporting Materials and Methods

Calibration of the rapid freeze-quench (RFQ) device from BioLogic

The binding of azide (N_3^-) to ferric myoglobin (Mb) (Eq. 1) is considered a standard reaction for the calibration of freeze-quench setups combined with EPR spectroscopy, due to its known kinetics and the easy-to-follow spin state change from the free ferric Mb (a high-spin iron(III) system with S = 5/2) to the azide-bound low-spin (S = 1/2) form.[1]

$$N_3^- + \text{Mb(High-Spin)} \rightarrow \text{Mb(Low-Spin)-}N_3 \qquad \text{[Eq. 1]}$$

In this work we adapted the method decribed by Pievo *et. al.*[2] to our system. More specifically, solutions of myoglobin from horse skeletal muscle (Sigma Aldrich) and sodium azide were prepared at a concentration of 1 mM and 5 mM, respectively, in order to work with a 5-fold molar excess of azide with respect to myoglobin. Lyophilized myoglobin was dissolved directly in 100 mM Tris-HCl buffer, pH 7. Sodium azide salt was dissolved in MilliQ water. Myoglobin and azide solutions were loaded on the freeze-quench syringes and samples at different time points were collected. The freezing bath consisted of cold isopentane at a temperature of -122 ± 3 °C. For each time point, 3 replicates were collected by mixing 70 µL of the myoglobin solution with 70 µL of the azide solution (final concentrations of myoglobin and azide being 0.5 mM and 2.5 mM, respectively).

The relative amounts of high-spin and low-spin myoglobin were assessed by CW X-band EPR spectroscopy. Measurements were performed at 9.95 ± 0.05 K under non-saturating conditions. The obtained spectra are plotted in Figure S4A, while the results of the calibration calculation are depicted in Figure S4B, where the X-axis represents the apparent reaction time (ms), defined as:

$$\text{Apparent reaction time} = \text{ageing time} + \text{flying time}$$

The ageing time is given by the volume of the Ejection Delay Line (EDL) which immediately follows the mixing chamber, divided by the flow rate, which can be set from the BioLogic BIOKINE software. The flying time is calculated by knowing the flow rate, the diameter of the exit nozzle (0.5 mm in the current setup) and the distance between the nozzle and the surface of the freezing bath (4.0 cm in the current setup). The Y-axis represents the ln(Y_t), where Y_t is defined as follows, according to Pievo *et. al.*[2]:

$$\frac{[HS]_0}{[HS]_t} = \frac{R_t}{R_t + \lambda} \qquad \text{[Eq. 2]}$$

where

$$R_t = \frac{(I_{HS})_t}{(I_{LS})_t} \qquad \text{[Eq. 3]}$$

and

$$\lambda = \frac{(I_{HS})_0}{(I_{LS})_\infty} \qquad \text{[Eq. 4]}$$

The parameter λ was calculated by measuring the CW X-band EPR spectra of a sample of Mb at pH 7.0 in absence of azide (resting state, 100 % high-spin) and a sample of Mb in presence of a 5-fold molar excess of NaN_3 which was left reacting for 5 seconds and then flash frozen in liquid nitrogen (reacted species, 100 % low-spin). The relative intensities were calculated, for each time points and for the two extremes of the reaction, as the ratio between the peak-to-peak distance of the low-field feature of high-spin Mb and the peak-to-peak distance of the g_y feature of low-spin Mb-N_3, all taken as absolute positive values. The intercept on the X-axis gives an apparent negative time which corresponds to the freezing time, a variable difficult to estimate otherwise, since it depends on several physical factors including the heat transfer coefficients between the sample and the cryogenic bath as well as the sample particles size and shape (and in turn the extension of the surface in contact with cryogenic liquid).[2] In

conclusion, to obtain the real quenching time of the reaction, the freezing time obtained by the calibration has to be added to the apparent reaction time. In our setup, we estimated a freezing time of about ~ 50 ms.

Density functional theory (DFT)

Spin-unrestricted density functional theory (DFT) calculations were performed using the ORCA package.[3,4] To mimic the solvent effect the COSMO model for water was used.[5] For the geometry optimizations of $ClO_2^{\bullet}$, the Becke-Perdew density functional (BP86)[6,7] was used. The Ahlrichs split-valence plus polarization (SVP) basis set was used for O atoms.[8] The Ahlrich (2df,2pd) polarization functions were obtained from the TurboMole basis set library as implemented in ORCA. For the Cl atom the doubly polarized triple-zeta (TZVPP) (Ahlrichs, unpublished) basis set was used. The energy was converged to 1×10^{-8} Hartree (Eh) and the tolerances of convergence in the geometry optimization were 3×10^{-4} Eh/Bohr for the gradient and 5×10^{-6} Eh for the total energy. For the single point calculations of the EPR parameters of the radical, d the PBE0 functional[9] was used in combination EPR-II[10] for oxygen and TZVPP for chlorine.

References

(1) Nami, F., Gast, P., and Groenen, E. J. J. (2016) Rapid freeze-quench EPR spectroscopy: Improved collection of frozen particles. *Appl. Magn. Res. 47*, 643–653.

(2) Pievo, R., Angerstein, B., Fielding, A. J., Koch, C., Feussner, I., and Bennati, M. (2013) A rapid freeze-quench setup for multi-frequency EPR spectroscopy of enzymatic reactions. *ChemPhysChem 14*, 4094–4101.

(3) Neese, F. (2001) Prediction of electron paramagnetic resonance *g* values using coupled perturbed Hartree–Fock and Kohn–Sham theory. *J. Chem. Phys. 115*, 11080.

(4) Neese, F. (2003) Metal and ligand hyperfine couplings in transition metal complexes: The effect of spin–orbit coupling as studied by coupled perturbed Kohn–Sham theory. *J. Chem. Phys. 118,* 3939.

(5) Sinnecker, S., Rajendran, Klamt, A., Diedenhofen, M. and Neese, F. (2006) Calculation of solvent shifts on electronic *g*-tensors with the conductor-like screening model (COSMO) and its self-consistent generalization to real solvents (direct COSMO-RS). *J. Phys. Chem. A 110*, 2235.

(6) Perdew, J. P. (1986) Density-functional approximation for the correlation energy of the inhomogeneous electron gas. *Phys. Rev. B 33*, 8822.

(7) Becke, A. D. (1988) Density-functional exchange-energy approximation with correct asymptotic behaviour. *Phys. Rev. A. 38*, 3098.

(8) Schäfer, A., Horn, H. and Ahlrichs, R. (1992) Fully optimized contracted Gaussian basis sets for atoms Li to Kr. *J. Chem. Phys. 97*, 2571.

(9) Adamo, C. and Barone, V. (1999) Toward reliable density functional methods without adjustable parameters: The PBE0 model. *J. Chem. Phys. 110*, 6158.

(10) Barone, V. (1995) in Recent Advances in Density Functional Methods (Part I, Ed. by D. P. Chong) World Scientific Publ. Co., Singapore, p 287.

Supporting Tables

Table S1. Comparison between the DFT-computed g and ^{35}Cl hyperfine values for $ClO_2^{\bullet}$ (see details of computation in the supporting Materials and Methods) and the parameters used to simulate the EPR spectrum in Figure S4D. The experimental error is 1 on the last digit.

	g_x	g_y	g_z	$^{Cl}A_x$ (MHz)	$^{Cl}A_y$ (MHz)	$^{Cl}A_z$ (MHz)
DFT	2.0019	2.0133	2.0174	200	-40	-31
experiment	2.002	2.011	2.017	212	-36	-21

Supporting Figures

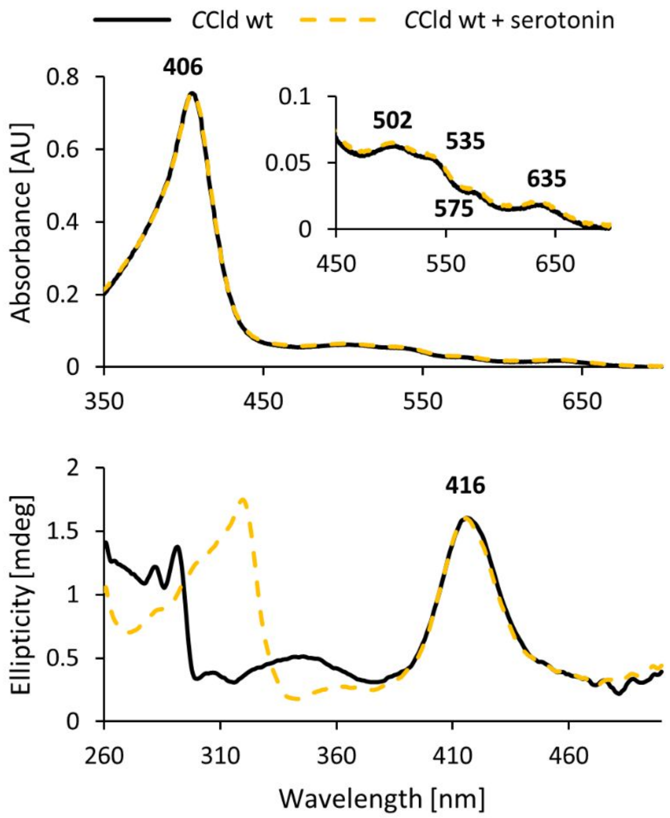

Figure S1. Impact of serotonin on the UV-vis- and electronic circular dichroism spectral features of wild-type *C*Cld at pH 7.0. Presence and absence of serotonin are represented by yellow dashed and black solid lines. For better visualization the inset in the top panel depicts the Q- and CT-band region (450-750 nm) in more detail. Enzyme and serotonin concentration: 10 µM and 10 mM, respectively. Note that the ECD signals in the far- and near-UV region of the *C*Cld + serotonin sample represent the intrinsic ellipticity of serotonin.

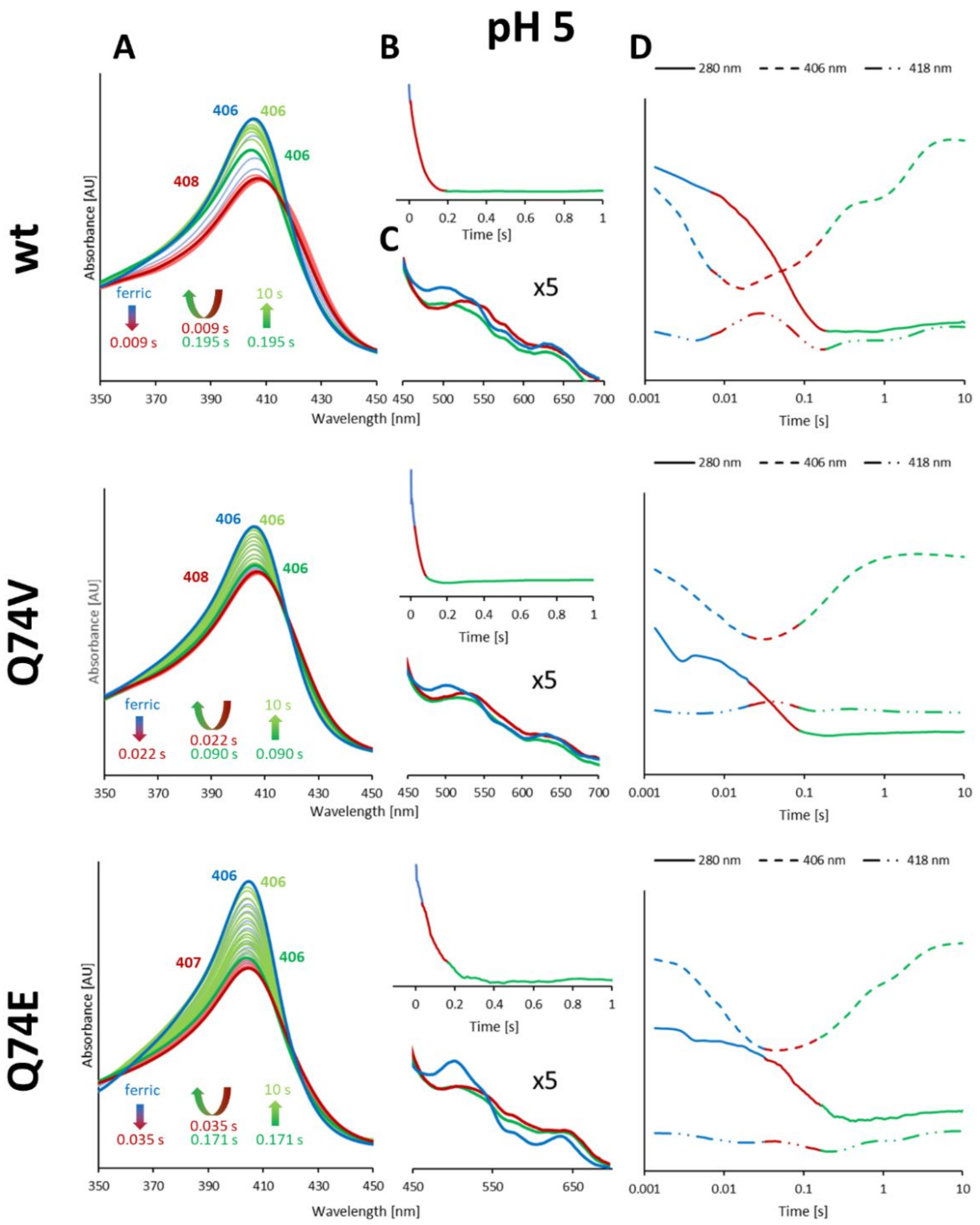

Figure S2. Reaction of wild-type *C*Cld and the variants Q74V and Q74E with ClO_2^- at pH 5.0. Reactions were followed by conventional stopped-flow spectroscopy. Final concentrations: 1.5 µM enzyme, 500 µM chlorite. (A, C) Interconversion of redox intermediates during reaction. The spectrum of the ferric protein as well as the spectral changes in the first fast phase of the reaction (*i.e.* Compound I formation, red bold spectrum) are shown in blue. The second phase, *i.e.* formation of Compound II/Compound I* is shown in red. The resulting species that dominates during chlorite degradation is shown in bold green. Spectral intermediates representing the slow final conversion back to the ferric resting state are displayed in green. (B) Chlorite degradation monitored by loss of absorbance at 280 nm. Colour code corresponds to that of (A). (D) Time traces reflecting chlorite degradation (280 nm, solid line), formation of Compound I and resting state (406 nm, dashed line) as well as formation and conversion of Compound II/Compound I* (418 nm, dash-dotted line). The x-axis is shown in logarithmic scale to have a better overview on the whole reaction.

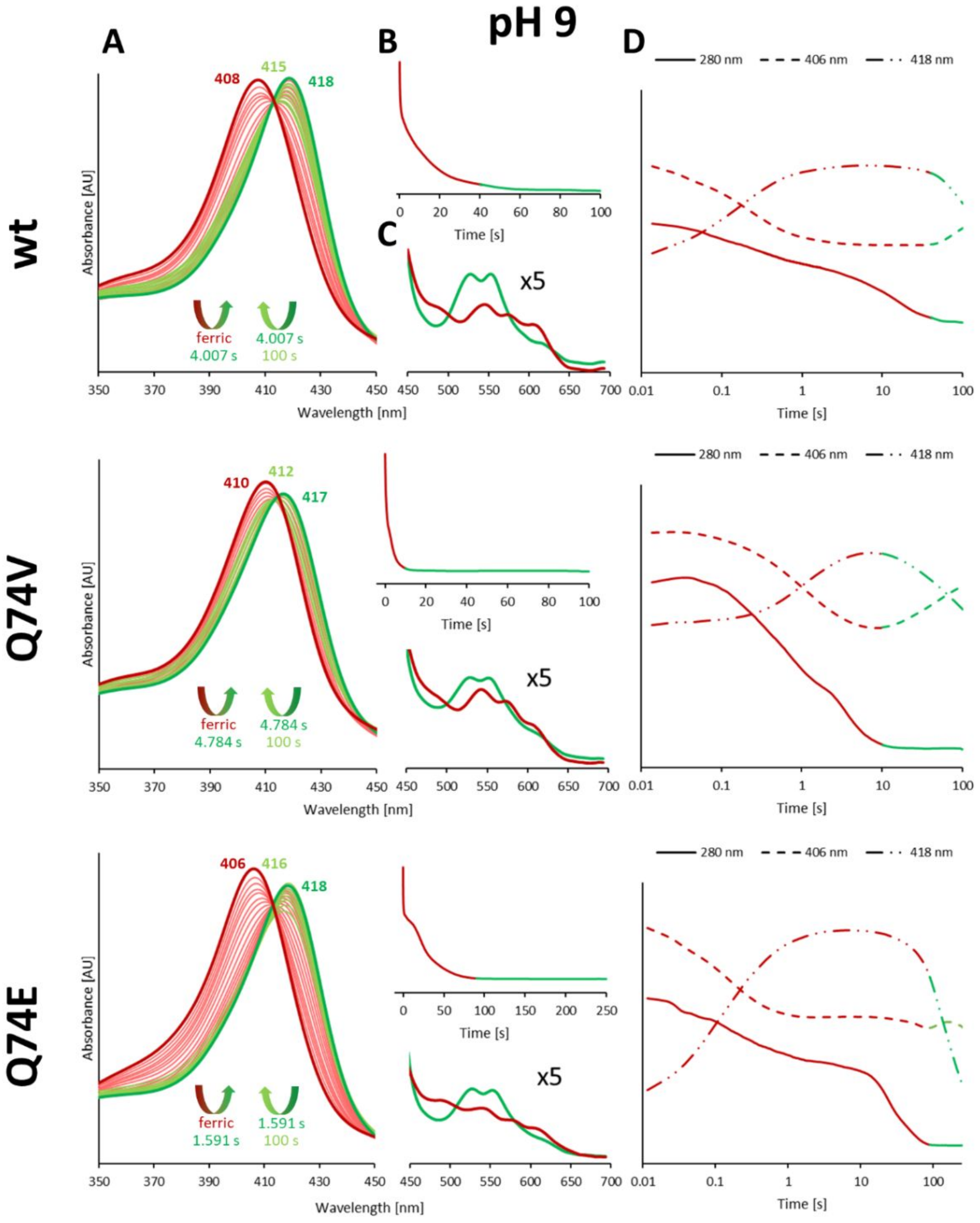

Figure S3. Reaction of wild-type *C*Cld and the variants Q74V and Q74E with ClO_2^- at pH 9.0. Reactions were followed by conventional stopped-flow spectroscopy. Final concentrations: 1.5 µM enzyme, 500 µM chlorite. (A, C) Interconversion of redox intermediates during reaction. The spectrum of the ferric protein (red bold spectrum) is converted to a species with Soret maximum at 418 nm (Compound II/Compound I* depicted in bold green). This species dominates during chlorite degradation and, finally, slowly converts to the resting state. (B) Chlorite degradation monitored by loss of absorbance at 280 nm. Colour code corresponds to that of (A). (D) Time traces reflecting chlorite degradation (280 nm, solid line), formation of Compound I and resting state (406 nm, dashed line) as well as formation and conversion of Compound II/Compound I* (418 nm, dash-dotted line). The x-axis is shown in logarithmic scale to have a better overview on the whole reaction.

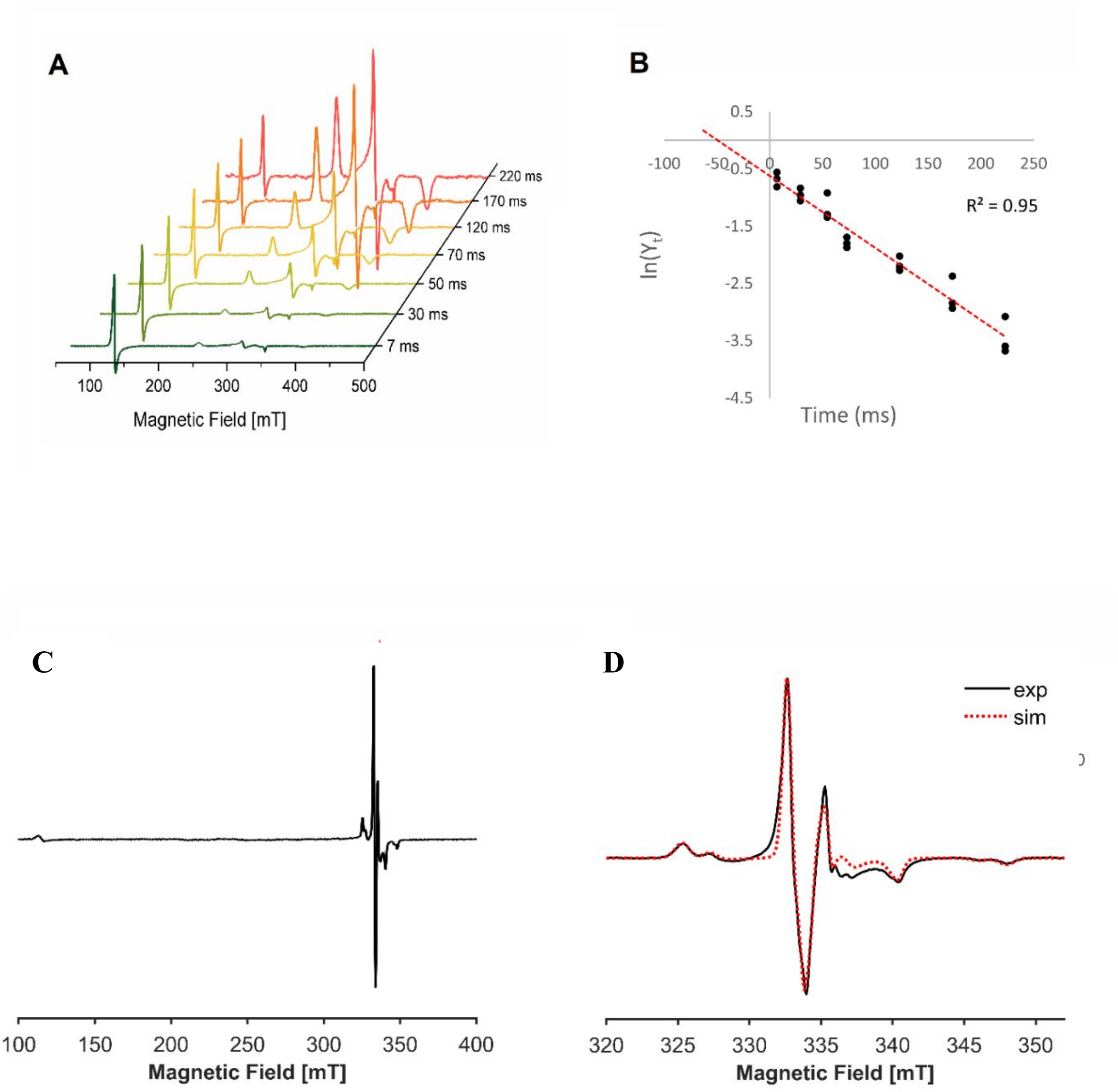

Figure S4. Calibration of the rapid freeze-quench device from BioLogic (A, B) and CW X-band EPR spectrum of wild-type *C*Cld in presence of a 300-fold molar excess of chlorite at pH 5.0 (C, D). (A) Calibration of the rapid freeze-quench device from BioLogic. Spectra of 0.5 mM myoglobin from horse skeletal muscle in presence of 2.5 mM sodium azide in 50 mM Tris-HCl, pH 7.0, where the binding reaction was quenched at different time points (apparent reaction time). The spectra were recorded at 10 K, with a MW power of 0.1 mW and a modulation amplitude of 1 mT. The intensities are normalized at the $g^{eff} \sim 6$ peak to better visualize the increasing formation of the low-spin azide-bound form; (B) Calibration curve obtained from the experiment shown in (A), where the X-axis represents the apparent reaction time (ms), while the Y-axis values are calculated as described in equations 1-4 presented in Supplementary information. The intercept on the X-axis gives the freezing time as a negative value, which has to be added to the apparent reaction time to calculate the real quenching time. (C, D) CW X-band EPR spectrum of 0.1 mM wild-type *C*Cld in presence of a 300-fold molar excess of chlorite (30 mM) at pH 5.0. The sample was prepared by RFQ (quenching time of ~ 60 ms) and the spectrum was recorded at 10 K, with a MW power of 1 mW and a modulation amplitude of 1 mT. (C) Full spectrum showing a minor feature at 115 mT representing a residual resting state signal; D) Zoom into the high field region of spectrum in (C) (solid black) and the corresponding simulation (dashed red).

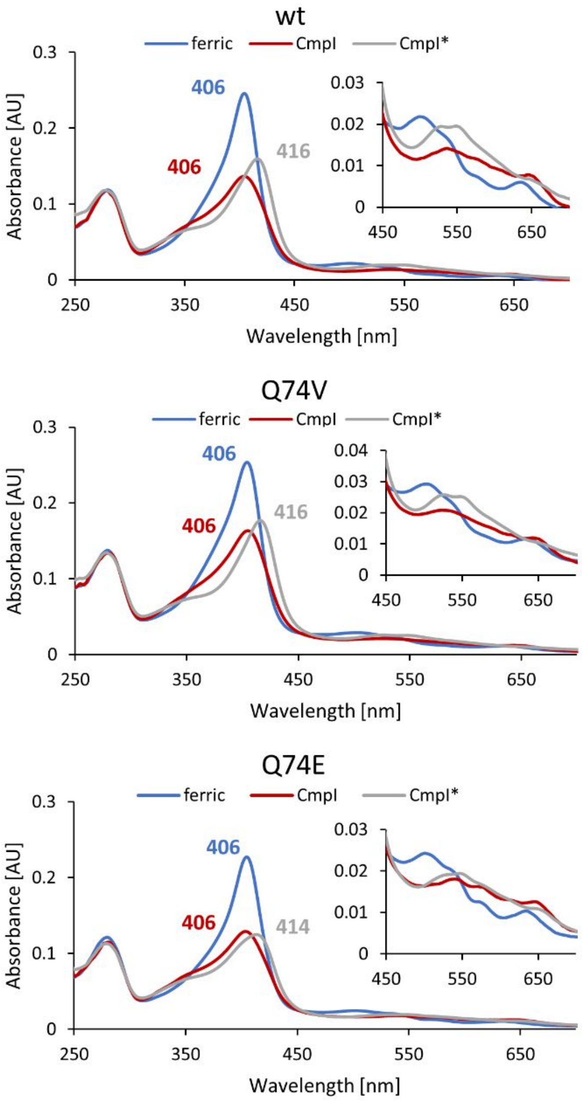

Figure S5. Interconversion of spectral features of wild-type *C*Cld and the variants Q74V and Q74E mediated by hypochlorite at pH 5.0. Conditions: 3 µM enzyme, 30 µM hypochlorite. Red spectra depict fully formed Compound I (50 ms: Q74V, 75 ms: wild-type *C*Cld, Q74E), whereas grey spectra represent the final spectrum (Compound I*) recorded after 4 s.

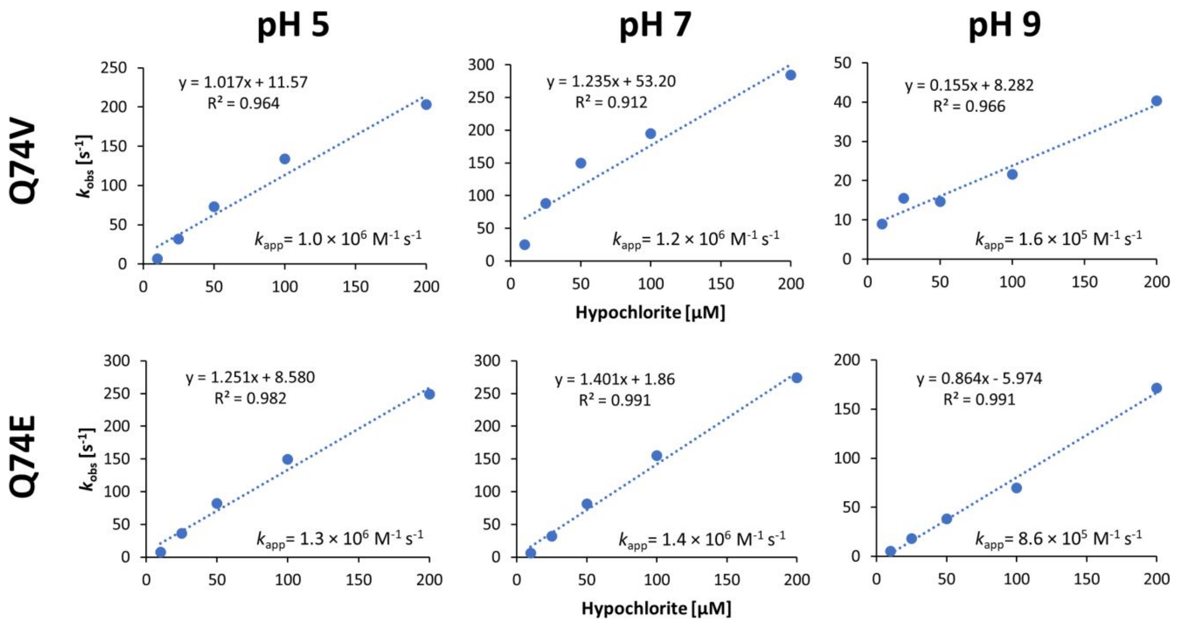

Figure S6. Kinetics of Compound I formation of the *C*Cld variants Q74V and Q74E at pH 5.0, 7.0 and 9.0. Linear plots of k_{obs} *versus* hypochlorite concentration (10 – 200 µM HOCl) used for calculation of apparent second-order rate constant k_{app} of Compound I formation. k_{obs} values were obtained from single exponential fitting the respective time traces. Enzyme concentration: 1.5 µM.

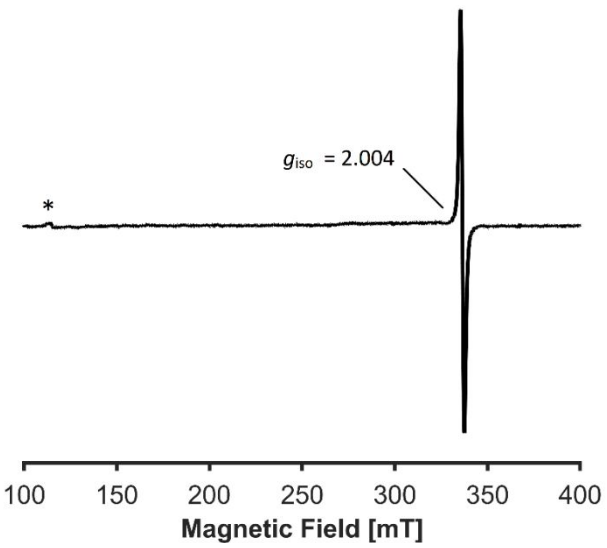

Figure S7. CW X-band EPR spectrum of 0.25 mM wild-type *C*Cld in presence of a 10-fold molar excess of hypochlorite (2.5 mM) at pH 5.0. The sample was prepared by RFQ (quenching time of ~ 60 ms) and the spectrum was recorded at 10 K, with a MW power of 0.2 mW and a modulation amplitude of 1 mT. The asterics indicate the presence of a residual high-spin signal corresponding to unreacted *C*Cld.

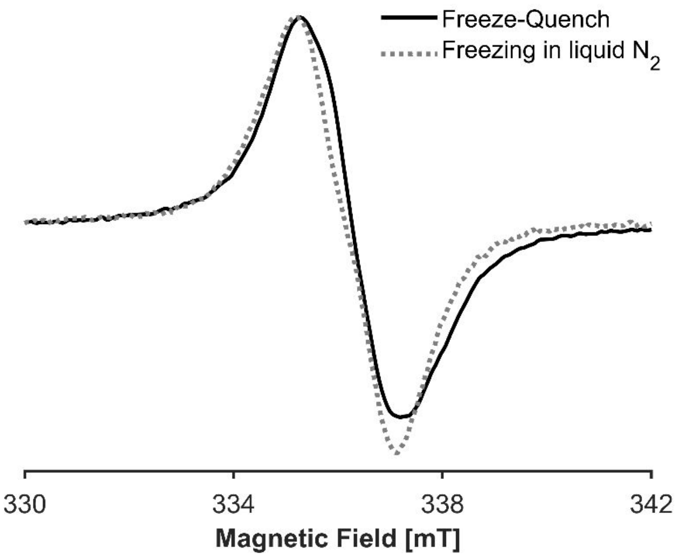

Figure S8. CW X-band EPR spectra of 0.25 mM wild-type *C*Cld in presence of a 10-fold molar excess of hypochlorite (2.5 mM) at pH 5.0 obtained by RFQ (solid black) and N_2 flash-freezing (dashed grey). The spectra were recorded at 10 K, with a MW power of 0.2 mW and 0.1 mW for the RFQ and the N_2 flash-frozen samples, respectively, and a modulation amplitude of 0.2 mT in both cases. The intensities are normalized to the maximum positive peak to highlight differences in lineshape.

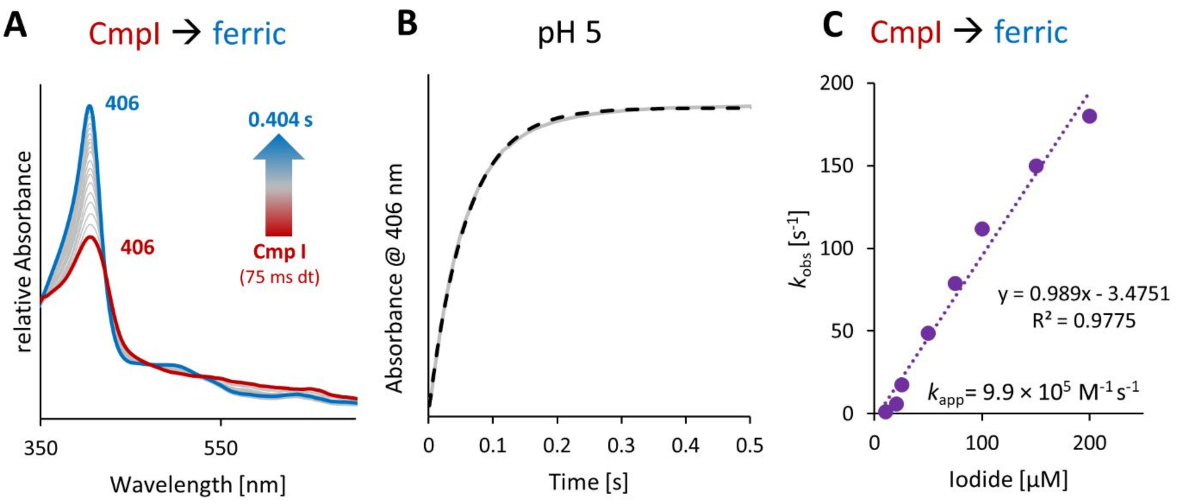

Figure S9. Reaction of wild-type *C*Cld Compound I with iodide at pH 5.0 (A) Spectral changes of reaction of Compound I (red spectrum), formed by mixing 1.5 µM wild-type ferric *C*Cld with a 10-fold excess of hypochlorite, with iodide at pH 5.0 followed by sequential stopped-flow spectroscopy. Delay time: 75 ms, at pH 5.0. The resulting ferric resting state is shown in blue. (B) Typical time trace at 406 nm and single exponential fit, shown as solid grey and dashed black lines, respectively. (C) Corresponding linear plot of k_{obs} *versus* iodide concentration of the reaction from Compound I directly back to the ferric resting state.

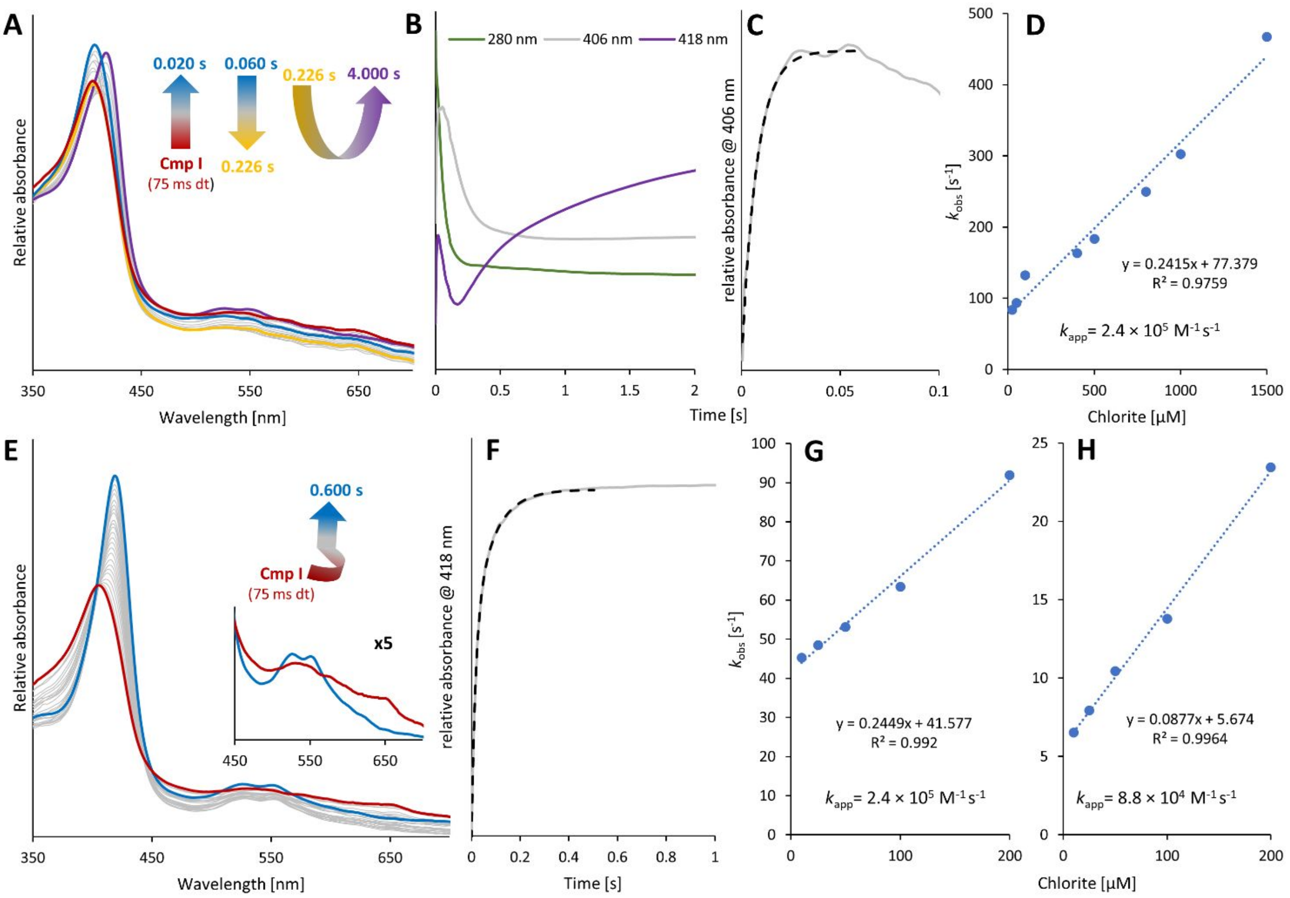

Figure S10. Reaction of wild-type *C*Cld Compound I with chlorite at pH 5.0 and 7.0 (A) Spectral changes of reaction of Compound I (red spectrum), formed by mixing 1.5 µM wild-type ferric *C*Cld with a 10-fold excess of hypochlorite, with chlorite at pH 5.0 followed by sequential stopped-flow spectroscopy. Delay time: 75 ms, at pH 5.0. (B) Important time traces of the reaction of Compound I with chlorite: time trace at 280 nm is representing the chlorite degradation. (C) Typical time trace at 406 nm and single exponential fit, shown as solid grey and dashed black lines. (D) Corresponding linear plot of k_{obs} *versus* chlorite concentration at pH 5.0. (E) Spectral changes of reaction of Compound I (red spectrum), formed by mixing 1.5 µM wild-type ferric *C*Cld with a 10-fold excess of hypochlorite, with chlorite at pH 7.0 followed by sequential stopped-flow spectroscopy. Delay time: 75 ms, at pH 5.0. (F) Typical time trace at 418 nm and double exponential fit, shown as solid grey and dashed black lines. (G, H) Corresponding linear plots of k_{obs} *versus* chlorite concentration of the first-(G) and the second phase of the reaction (H).

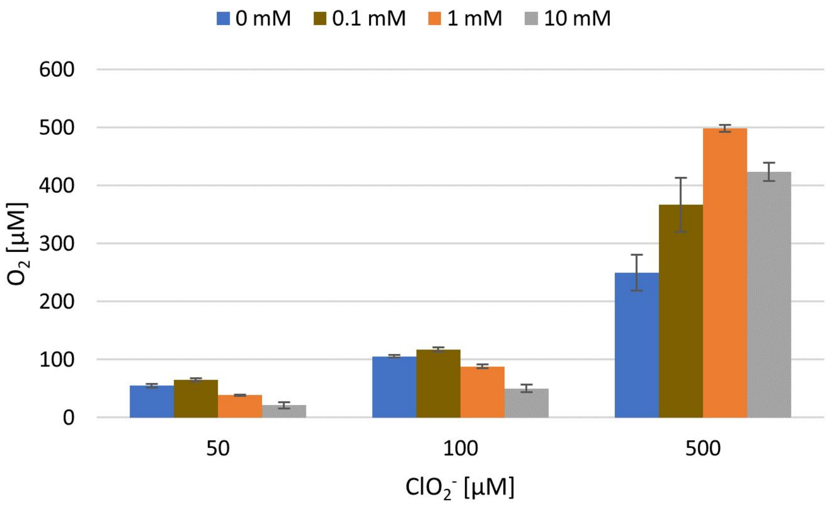

Figure S11. Impact of serotonin on chlorite degradation and O_2 production at pH 7.0. Dioxygen release was followed polarorophically. Reaction conditions: wild-type *C*Cld: 20 nM, chlorite: 50, 100 and 500 µM; serotonin: 0, 100 µM, 1000 µM, 10000 µM. Measurements were done in triplicates.

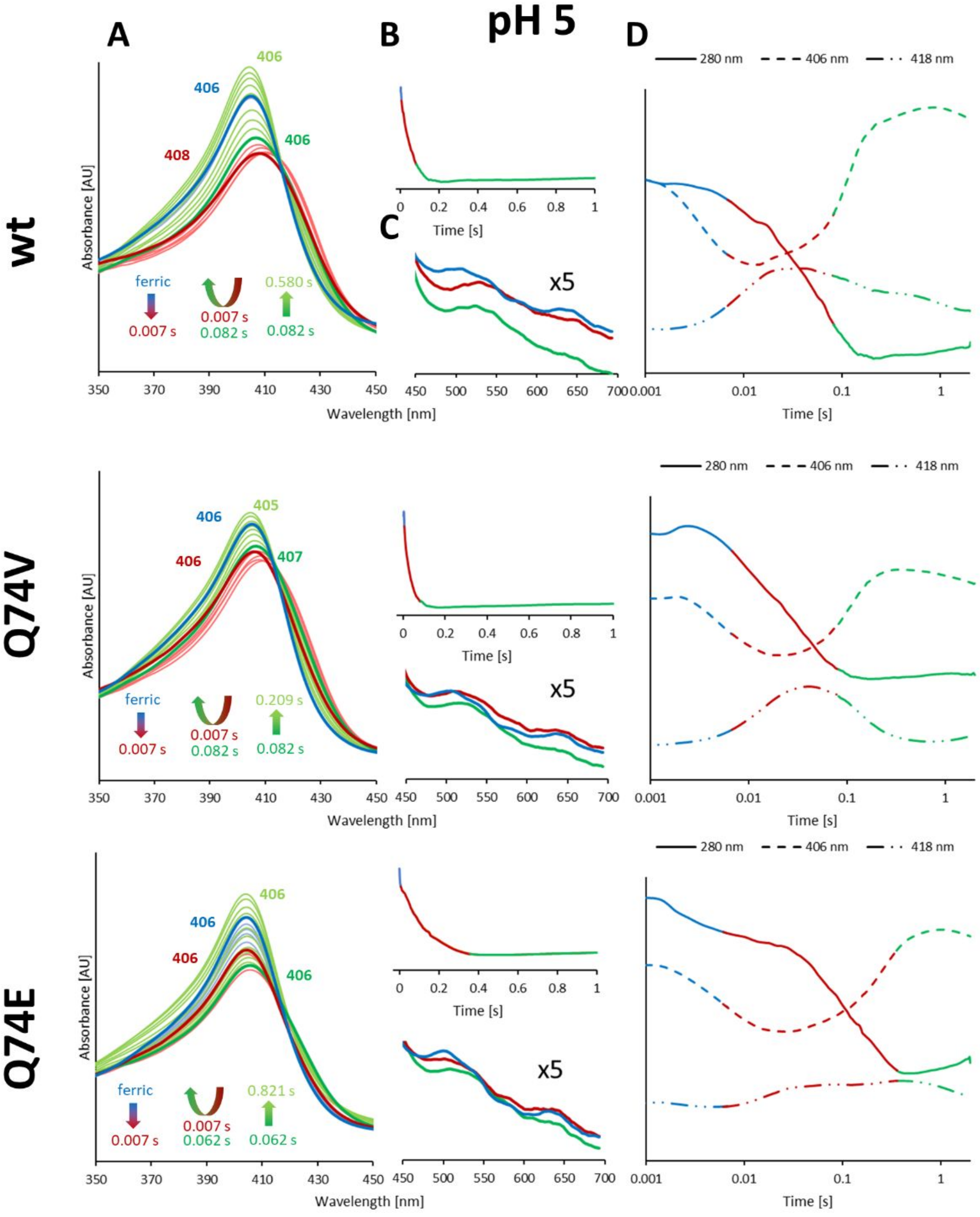

Figure S12. Reaction of wild-type *C*Cld and the variants Q74V and Q74E with ClO_2^- in the presence of serotonin at pH 5.0. Reactions were followed by conventional stopped-flow spectroscopy. Final concentrations: 1.5 µM enzyme, 500 µM chlorite, 100 µM serotonin. (A, C) Interconversion of redox intermediates during reaction. The spectrum of the ferric protein as well as the spectral changes in the first fast phase of the reaction (i.e. Compound I formation, red bold spectrum) are shown in blue. The second phase, i.e. formation of Compound II/Compound I* is shown in red. The resulting oxoiron(IV) species that dominates during chlorite degradation is shown in bold green. Spectral intermediates representing the slow final conversion to the ferric resting state are displayed in green. (B) Chlorite degradation monitored by loss of absorbance at 280 nm. Colour code corresponds to that of (A). (D) Time traces reflecting chlorite degradation (280 nm, solid line), formation of Compound I and resting state (406 nm, dashed line) as well as formation and conversion of Compound II/Compound I* (418 nm, dash-dotted line). The x-axis is shown in logarithmic scale to have a better overview on the whole reaction.

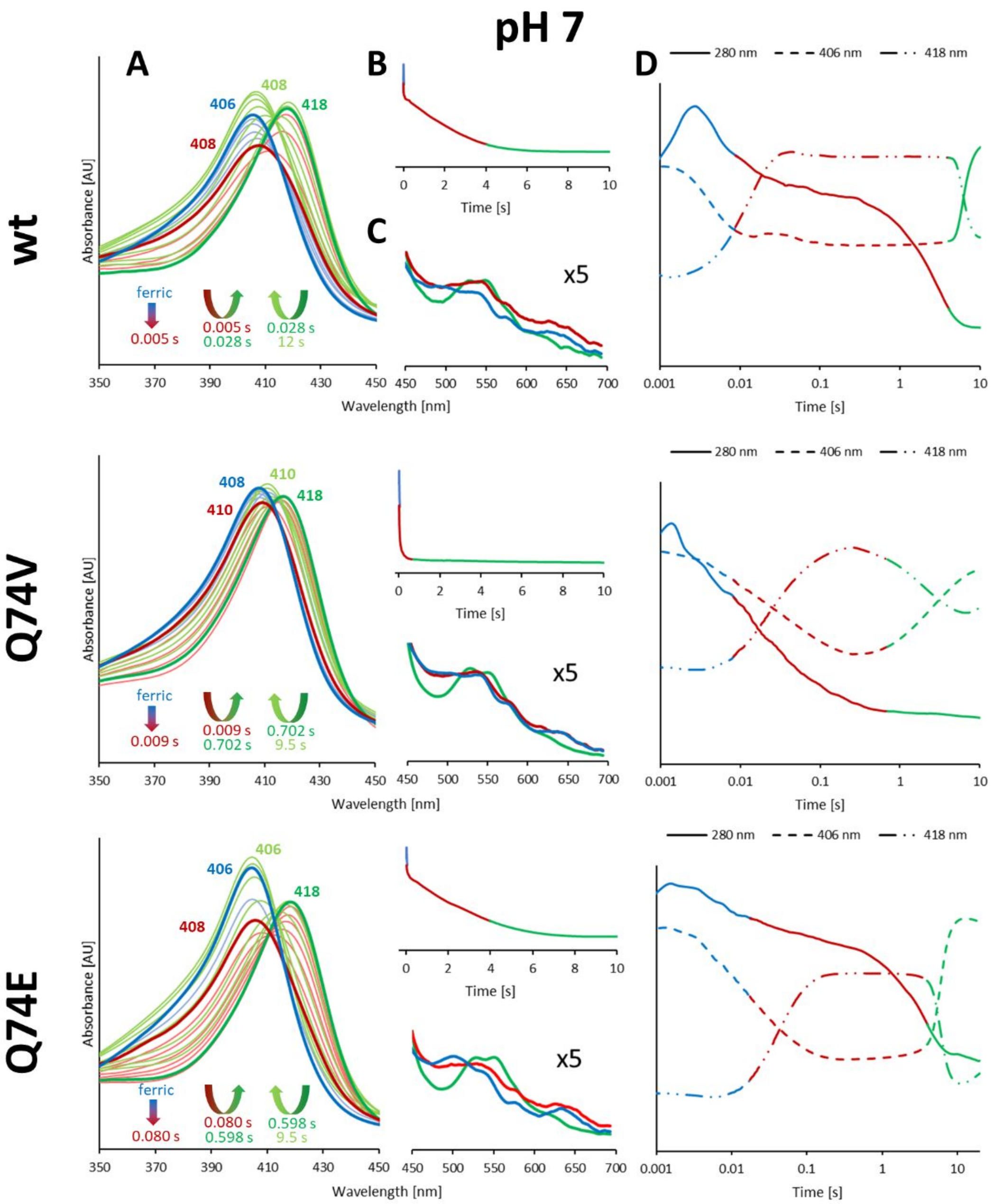

Figure S13. Reaction of wild-type *C*Cld and the variants Q74V and Q74E with ClO_2^- in the presence of serotonin at pH 7.0. Reactions were followed by conventional stopped-flow spectroscopy. Final concentrations: 1.5 µM enzyme, 500 µM chlorite, 100 µM serotonin. (A, C) Interconversion of redox intermediates during reaction. The spectrum of the ferric protein as well as the spectral changes in the first fast phase of the reaction (i.e. Compound I formation, red bold spectrum) are shown in blue. The second phase, i.e. formation of Compound II/Compound I* is shown in red. The resulting oxoiron(IV) species that dominates during chlorite degradation is shown in bold green. Spectral intermediates representing the slow final conversion to the ferric resting state are displayed in green. (B) Chlorite degradation monitored by loss of absorbance at 280 nm. Colour code corresponds to that of (A). (D) Time traces reflecting chlorite degradation (280 nm, solid line), formation of Compound I and resting state (406 nm, dashed line) as well as formation and conversion of Compound II/Compound I* (418 nm, dash-dotted line). The x-axis is shown in logarithmic scale to have a better overview on the whole reaction.

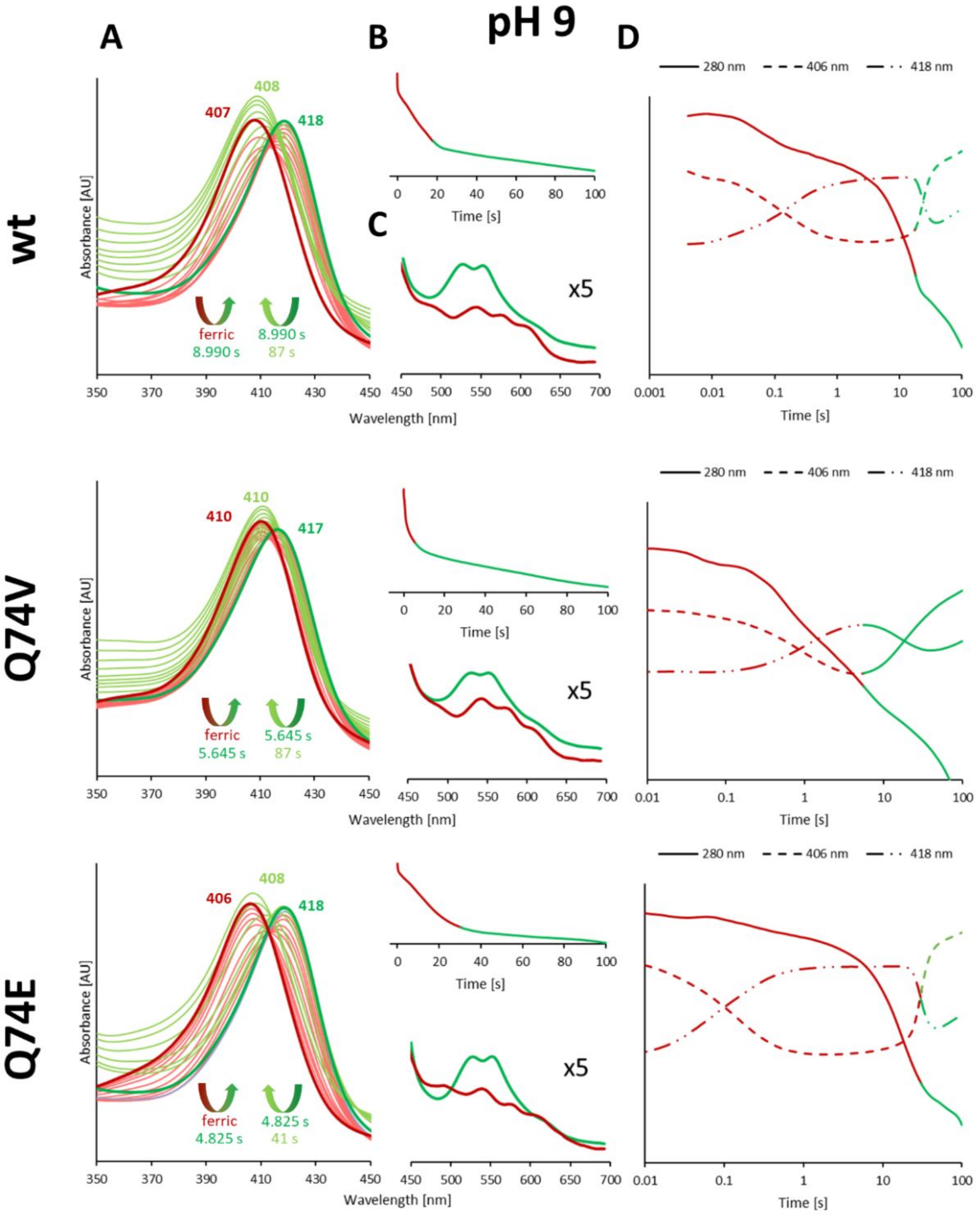

Figure S14. Reaction of wild-type *C*Cld and the variants Q74V and Q74E with ClO_2^- in the presence of serotonin at pH 9.0. Reactions were followed by conventional stopped-flow spectroscopy. Final concentrations: 1.5 µM enzyme, 500 µM chlorite, 100 µM serotonin. (A, C) Interconversion of redox intermediates during reaction. The spectrum of the ferric protein (red bold spectrum) is converted to a species with Soret maximum at 418 nm (Compound II/Compound I* depicted in bold green). This species dominates during chlorite degradation and, finally, converts to the ferric resting state. (B) Chlorite degradation monitored by loss of absorbance at 280 nm. Colour code corresponds to that of (A). (D) Time traces reflecting chlorite degradation (280 nm, solid line), formation of Compound I and resting state (406 nm, dashed line) as well as formation and conversion of Compound II/Compound I* (418 nm, dash-dotted line). The x-axis is shown in logarithmic scale to have a better overview on the whole reaction.
